# Self-Assembly of Organic Nanomaterials and Biomaterials: The Bottom-Up Approach for Functional Nanostructures Formation and Advanced Applications

**DOI:** 10.3390/ma13051048

**Published:** 2020-02-26

**Authors:** Domenico Lombardo, Pietro Calandra, Luigi Pasqua, Salvatore Magazù

**Affiliations:** 1 Consiglio Nazionale delle Ricerche, Istituto per i Processi Chimico-Fisici, 98158 Messina, Italy; 2 Consiglio Nazionale delle Ricerche, Istituto Studio Materiali Nanostrutturati, 00015 Roma, Italy; pietro.calandra@cnr.it; 3 Department of Environmental and Chemical Engineering, University of Calabria, 87036 Rende, Italy; luigi.pasqua@unical.it; 4 Dipartimento di Scienze Matematiche e Informatiche, Scienze Fisiche e Scienze della Terra, Università di Messina, 98166 Messina, Italy; smagazu@unime.it

**Keywords:** self-assembly nanomaterials, biomaterials, polymer nanomaterials, drug delivery, biotechnology, amphiphiles, block copolymers, hydrogels, biomolecules

## Abstract

In this paper, we survey recent advances in the self-assembly processes of novel functional platforms for nanomaterials and biomaterials applications. We provide an organized overview, by analyzing the main factors that influence the formation of organic nanostructured systems, while putting into evidence the main challenges, limitations and emerging approaches in the various fields of nanotechology and biotechnology. We outline how the building blocks properties, the mutual and cooperative interactions, as well as the initial spatial configuration (and environment conditions) play a fundamental role in the construction of efficient nanostructured materials with desired functional properties. The insertion of functional endgroups (such as polymers, peptides or DNA) within the nanostructured units has enormously increased the complexity of morphologies and functions that can be designed in the fabrication of bio-inspired materials capable of mimicking biological activity. However, unwanted or uncontrollable effects originating from unexpected thermodynamic perturbations or complex cooperative interactions interfere at the molecular level with the designed assembly process. Correction and harmonization of unwanted processes is one of the major challenges of the next decades and requires a deeper knowledge and understanding of the key factors that drive the formation of nanomaterials. Self-assembly of nanomaterials still remains a central topic of current research located at the interface between material science and engineering, biotechnology and nanomedicine, and it will continue to stimulate the renewed interest of biologist, physicists and materials engineers by combining the principles of molecular self-assembly with the concept of supramolecular chemistry.

## 1. Introduction

Materials self-assembly is a key strategy for the design and fabrication of nanostructured systems and has become a fundamental approach for the construction of advanced materials and their application in the fields of nanomaterials and biotechnology [1,2,3]. In nanomaterials self-assembly, the mutual interaction between disordered building blocks drive a material system toward the spontaneous formation of more ordered (or more organized) nano-structured systems. Main examples of the self-assembly method can be found in biomaterials, where the interaction of various macromolecular components and the integration of their actions allow the occurrence of highly specific functions of biological interest [4,5,6]. For example, the folding of a polypeptide chain within a protein or the nucleic acids conformational changes into a variety of functional forms are important examples of self-assembly processes involved in many biological functions [7].

The construction and optimization of functional materials at the atomic and molecular level require a structural control and investigation at the nano-scale and have stimulated a parallel development of instrumentation techniques and observational methods on those size scales. Moreover, the development of multifunctional nanostructures and biomaterials using the recent concepts of nanoarchitectonics utilizes advanced processes for the self-assembly, such as supramolecular chemistry and host–guest processes [7,8,9]. The reversibility of the noncovalent forces allows a dynamic switching of the structure and morphology of the nanostructures in response to various (internal and external) stimuli, thus providing additional flexibility for the design and fabrication of versatile smart materials and functional nano-devices [10,11,12]. Finally, the new structure and properties obtained by the precise engineering of chemical structures allow the modulation and control of morphology and the efficient use of noncovalent forces (and structure directing interactions) with the introduction of chirality, signal processing and recognition processes [7,13].

In this review we highlight the recent development of the self-assembly approaches with a focus on the main properties and recent applications. We present an organized overview, by analyzing the main parameters that sensitively influence the design of organic nanostructured systems, while putting into evidence challenges, limitations and emerging approaches in the various fields of nanotechology and biotechnology.

## 2. Common Features of the Self-Assembly Processes in Nanoscience

**Figure 1:**
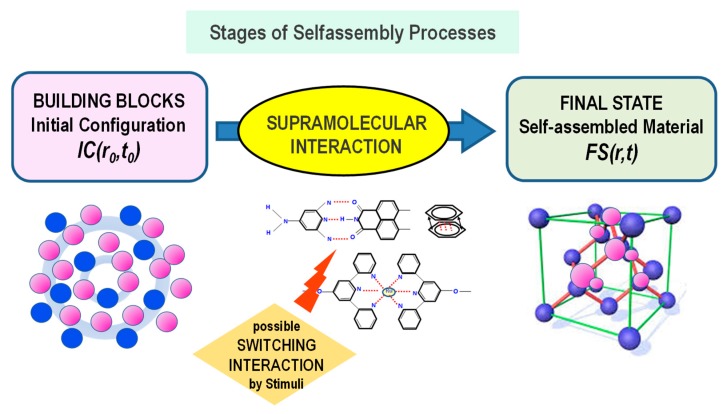
Conceptual scheme indicating the main stages of the self-assembly process in nanoscience.

The nanotechnology self-assembly processes are based on the employment of cooperative interactions between basic units (called building blocks) and allow a suitable control of the formation processes by which multicomponent systems evolve towards novel equilibrium states through a spontaneous organization process. These cooperative interactions are established within an initial configuration of (disordered) building blocks. The combination of cooperative (non-covalent) forces with the multi-functionality of building blocks provides an excellent strategy for the preparation of novel, advanced nanomaterials [14]. The various types of self-assembly processes of nanoscience have some common features that allow for the adoption of a conceptual scheme based on the following three stages: (Figure 1).

### 2.1. Initial State: Spatial Configuration of Basic Components (Building Blocks)

Self-assembly processes start with an ensemble of basic component units (atoms/molecules), with a given space distribution (initial configuration), which interact with each other [15,16,17]. These building blocks include a wide range of organic and inorganic macromolecules, with different masses, morphology, chemical compositions and functionalities (such as charge, polarizability, magnetic dipole, surface properties). Examples include hybrid units containing crystals, colloids, nanowires, lipid bilayers, carbohydrates, peptides, proteins and oligonucleotides [5,9,14].

### 2.2. (Supra) Molecular Interaction

The construction of advanced nanomaterials by the self-assembly process involves combination of a given number of driving supramolecular forces between the building blocks. These forces are generally non-covalent interactions, and include electrostatic forces, hydrophobic interaction, hydrogen bonding, van der Waals forces, host–guest recognition, metal coordination and π-π stacking interaction [14,17]. These forces, which are much less intense than the covalent bonds (400 kJ ·mol^−1^), make the system more flexible, and enable to preserve the reversibility of the self-assembled nanostructures. In Table 1, we report a summary of those soft interactions. The interacting forces often occur between different sites of the building blocks and may strongly depend on the environment conditions. Possible trigger effects caused by (internal/external) stimuli may activate a structural response of the system (stimuli responsive nanomaterials) [10,11]. Moreover, the interactions among the building blocks in their initial configuration must be weak enough (compared to thermal energies) to be broken if desired (i.e., reversible and controllable). Despite the involved weak interactions in nanomaterials self-assembly, their high number is enough to hold the various building blocks together and to ensure their chemico-physical stability within their specific micro-environment.

### 2.3. Final State: Formation of Functional Supramolecular Structures

The self-assembly processes terminate with the spontaneous formation of (reversible and/or controllable) organized structures. The process is characterized by a transition from some initial less ordered configuration (such as a dispersion of disordered macromolecules or aggregates) toward a final more favorable, ordered, self-assembled nanostructures (such as a condensed state or a crystal). This requires an energy minimum at the final equilibrium configuration, characterized by a higher order (i.e., a lower entropy) than the initial configuration of isolated component units, while its surroundings present in general a more disordered state (i.e., a higher entropy). The self-assembly process of multi-component systems can yield structures at several orders of magnitude in size. This characteristic arises from the variety of combinations of phase transitions of one or more constituents. Due to these controllable properties and the reversible nature of the generated nanostructures, the final nanomaterials are expected to have a high function/performance suitable for advanced nanotechnology application.

Finally, the physico-chemical properties of nanomaterials can be modified by changing their size, shapes (such as sphere, rod, hyperbranched, lamellar or layered structures), chemical compositions (organic, inorganic, hybrid or composites), and surface properties (surface charge and coating, functional groups, or targeting endgroups). In the following, we will describe the main features of the nanomaterials self-assembly.

The organic nanomaterials, which consist of carbon-based components, are characterized by a high versatile control of both chemical composition and morphology. We can identify two main typologies of organic building blocks, namely the traditional “head-and-tail” amphiphiles, and polymer-based building blocks.

## 3. Traditional Amphiphile Building Blocks: Micelles, Vesicles, Liquid Crystalline Nanostructures and Microemulsions

The self-assembly of amphiphilic molecules represents the precursor of the bottom-up methods in modern nanoscience, whereby the segregation of hydrophobic and hydrophilic regions is the primary interaction that drives the formation of various phases such as micellar aggregates, vesicles, lamellar structures, nano- and microemulsions and (lyotropic) liquid crystals. The formation of those phases depends on different parameters of the system, such as amphiphile shape, concentration, temperature and solution conditions [15,16,17,18,19].

The traditional head-and-tail amphiphiles are composed of an uncharged (or charged) hydrophilic portion (head) and one hydrophobic (or lipophilic) portion, such as the hydrocarbon chains (tail). In selective solvents (such as water), the amphiphiles promote a microphase separation between the dispersed phase and the solvent, with the formation of micelles-like aggregates. These aggregates can be detected through the measurement of a discontinuity in some properties of the system (such as the osmotic pressure, surface tension, turbidity, equivalent conductivity) at a specific concentration (critical micelle concentration-CMC) and at a given temperature (critical micelle temperature-CMT) [16,17]. It is worth mentioning that a number of investigations have evidenced the presence of a premicellar aggregation process characterized by the presence of aggregates at concentrations significantly lower than the CMC [20,21,22].

**Figure 2:**
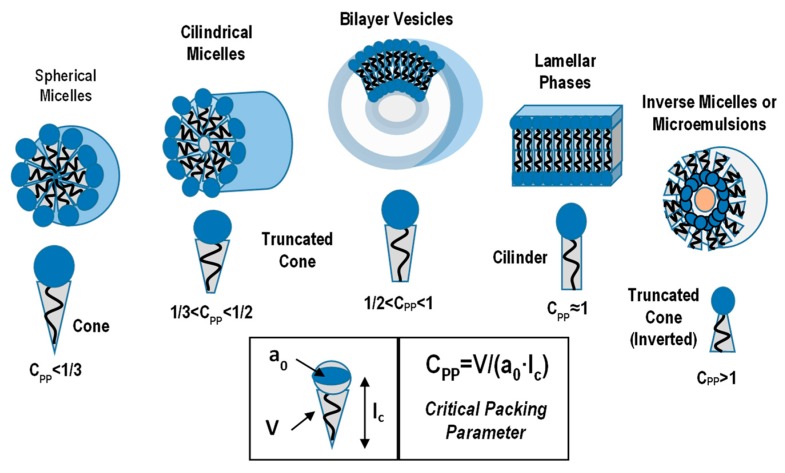
Amphiphile aggregates morphology and analysis of the critical packing parameter Cpp.

The prediction of the amphiphile aggregate morphology (Figure 2) can be performed by the analysis of the critical packing parameter C_pp_ = V_0_/A_mic_ l_c_, where V_0_ is the hydrophobic chains effective volume (core region), l_c_ is the maximum chain length (critical chain length) and A_mic_ is the effective hydrophilic head-group surface area (at aggregate–solution interface) [16,17,18]. With the increasing value of C_pp_, the amphiphilic aggregates pass from spherical (C_pp_ ≤ 1/3), to cylindrical (1/3 ≤ C_pp_ ≤ 1/2) and lamellar structure (C_pp_ = 1). Finally, vesicles are generally formed for 1/2 ≤ C_pp_ ≤ 1, which correspond to closed bilayer structures (sphere or ellipsoid) that have an internal cavity [16,17].

However, the amphiphile aggregates can be modulated to a wide range of other phases (such as rods, disks, ribbons, helix) or to ordered liquid crystalline morphologies by modifying certain control parameters, including the temperature, concentration, ionic strength and pH of the solution [23,24,25,26].

### 3.1. Micelles and Vesicles Nanostructures

**Figure 3:**
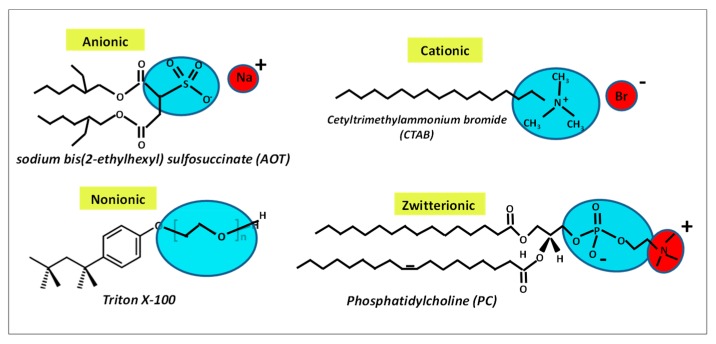
Example of traditional charged (anionic/cationic/zwitterionic) and uncharged (nonionic) amphiphilic molecules.

Micelles are the main aggregate morphologies that are formed in solution of a large variety of conventional ionic/nonionic surfactants [26,27,28]. Triton X-100 and the polyoxyethylene alkyl ethers, CnEm, (consisting m hydrophilic oxyethylene units Em and an alkyl chain with n methylene groups Cn) are among the most investigated nonionic amphiphiles. Moreover, a large variety of aggregates is obtained by the self-assembly processes of charged amphiphiles. For example, the anionic amphiphiles of sodium dodecyl sulphate (SDS), sodium bis-2-ethylhexyl sulphosuccinate (AOT), and the cationic cetyltrimethylammonium bromide (CTAB) amphiphiles are among the most investigated charged amphiphilic systems [15,16,17,18]. Finally, in zwitterionic amphiphiles, the headgroups have both a positive and a negative charge, as found in many (phospho-) lipid systems [16,27]. For example, phosphatidylcholine (PC) lipids consists of a double alkyl hydrophobic chain and a zwitterionic headgroup composed of a negatively charged phosphate which is separated from a positively charged nitrogen of choline unit. In aqueous solution lipid-based amphiphiles spontaneously self-assemble to form bilayer vesicles, which is the typical morphology found in bio-membranes. In Figure 3, we report an example of traditional charged (anionic/cationic/zwitterionic) and uncharged (nonionic) amphiphilic molecules.

**Figure 4:**
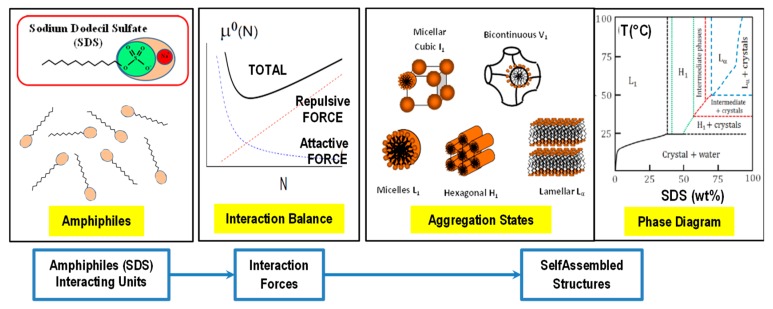
Main features of the self-assembly processes in a water solution of a traditional amphiphile. The phase diagram of the sodium dodecyl sulphate (SDS)/water system is taken from Reference [20].

It is worth pointing out that amphiphilic systems containing mixed surfactant components may induce complex synergetic effects, at given concentrations, that strongly influence the physico-chemical properties of their individual components, thus favoring the spontaneous formation of novel fascinating nanostructures, like mixed micelles, vesicles, ribbons, helix, fibrils and a large variety of extended networks with novel properties [28,29,30,31]. In Figure 4, we report the main stages of the self-assembly processes in a water solution of a traditional amphiphile.

### 3.2. Liquid Crystalline Nanostructures

At the higher amphiphile concentrations the anisotropic amphiphilic building blocks can spontaneously order themselves in a liquid crystalline (LCs) phase, characterized by an aggregation state which is intermediate between that of a liquid and a crystalline (solids) phase [32,33]. The formation of the lyotropic liquid crystalline phase is strongly influenced by the intensity of the repulsive forces between adjacent headgroups at the surfactant–water interface, the degree of contact between water and alkyl chain, and the conformational disorders of the alkyl chains.

In this scenario, amphiphilic molecules must arrange themselves to simultaneously minimize the steric hinderance and maximizing their polar–polar (and apolar–apolar) interactions [34,35,36]. Due to their low abundance in the systems, solvent molecules end up being confined in regions with the same polarity, while the amphiphiles form strong templating cages. New regions, characterized by the presence of liquid crystal phases, are generally formed [37,38]. It is obvious that the specific mixture of various amphiphiles can trigger the arising of emerging properties that are suitable for the construction of novel advanced devices in many diverse nanotechnology areas, such as displays, nanosensors and biotechnology applications [37,38]. If the amphiphiles are already in the liquid state, solvent molecules are no longer necessary, so the merits deriving from the amphiphiles characteristics are driven to the extreme [39]. The possibility to form new phases opens new doors: For example, the formation of nanoporous materials that have 1D/2D/3D channels (containing nanosized pores) based on the polymerizable liquid crystals can stimulate the development of novel applications such as adsorbents, filtration, ion exchange membranes and scaffolds for catalytic reactions [40].

The most remarkable challenges for the construction of these nanostructured systems include the control of pores (sizes and surface chemistry) as well as the integration of highly functional polymers and active compounds into the advanced nano-devices [41,42]. Liquid crystals are good materials for organic field effect transistors (OFETs), because of their good solution processability for the construction of (uniform) crystalline thin films by using a precursor of the liquid crystalline phase [41]. For example, a novel liquid crystalline (organic) semiconductor of 2-phenyl-7-decyl[1] benzothieno(3,2-b)[1]benzothiophene (Ph-BTBT-10) has been recently constructed, starting from a highly ordered (Smectic E) liquid crystal phase. The LCs semiconductor shows also high thermal durability and high solubility. Those features can be employed for the development of transistor devices that have a high mobility (over 10 cm^2^ Vs^−1^, by means of a thermal annealing at the temperature of T = 120 °C for 5 min) [41].

**Figure 5:**
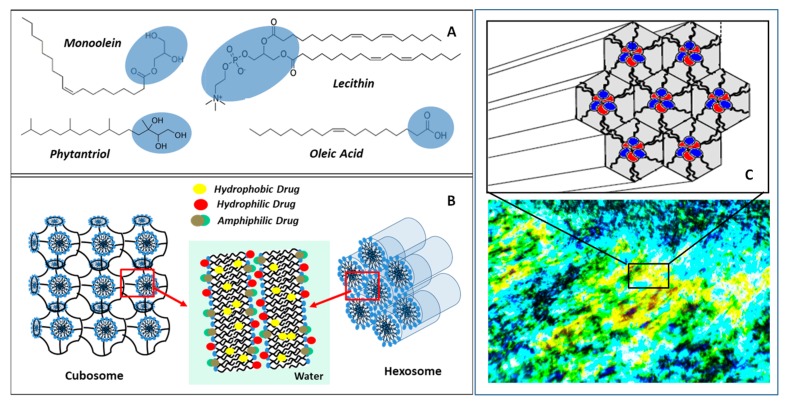
Some of the most commonly used amphiphiles for the construction of liquid crystalline (LCs) nanostructures (**A**). Typical bicontinuous gel-like cubic and inverted hexagonal phases contain hydrophilic and hydrophobic compartments which can encapsulate hydrophilic or lipophilic guest compounds (drugs) (**B**). Polarized optical micrographs of an equimolar bis(2-ethylhexyl) phosphoric acid/n-octylamine mixture (**C**), and the 2D hexagonal molecular rearrangement at the basis of the LCs structure (top).

In Figure 5A, we report some of the commonly used amphiphiles for the construction of LCs nanostructures. Monoolein is a commonly used non-toxic and biocompatible, polar lipid, which has low water solubility, that forms different LCs phases in excess water [42]. The lipid phytantriol was shown to generate cubic LCs, that are not susceptible to esterase catalyzed hydrolysis, thus providing an additional colloidal stability to the formed phases. Moreover, phytantriol is largely employed as an active compound in the cosmetics applications for the improvement of the moisture retention [33,43].

Due to their ability to include a vast array of lipophilic and hydrophilic molecules, LCs nanostructures are particularly interesting for drug delivery and for tissue engineering applications [43,44]. More specifically, thermodynamically stable liquid crystalline nanocarriers (LCNs) are formed upon the water dispersion of amphiphiles molecules with lyotropic lipids (such as unsaturated monoglycerides, phospholipids, glycolipids). These organized nanostructures are characterized by a bicontinuous cubic, inverted hexagonal or sponge phases, and contain hydrophilic and hydrophobic compartments which can efficiently encapsulate hydrophilic or lipophilic drugs or guest macromolecules (Figure 5B). In Figure 5C, we report the hexagonal nanostructure (polarized optical micrographs) obtained by the self-assembly of an equimolar surfactant-based mixture of bis(2-ethylhexyl) phosphoric acid/n-octylamine systems, and the corresponding 2D hexagonal molecular rearrangement at the basis of the LCs structure (top).

**Scheme 1:**
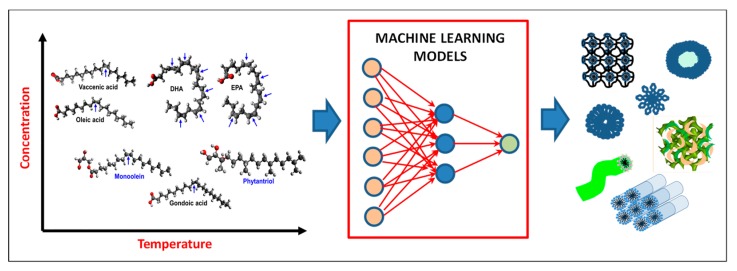
Sketch of main features of the machine learning model approach. Adapted from reference [45].

Recently, a *machine learning approach* has been used to predict the self-assembly of LCNs consisting of a base lipid, (composed of monoolein, or phytantriol) and a variety saturated (10 different types) and unsaturated (20 different types) fatty acids at different concentrations (fatty acid/lipid ratios), and temperatures [45]. For each of the seven mesophases identified, two separate models have been developed, i.e., multiple linear regression (MLR) and Bayesian regularized artificial neural networks (ANNs). The experimental data, acquired by high throughput characterization techniques, were used to train the (neural networks) machine. The models were capable of interpolating the experimental data (with different combinations of fatty acids) at temperatures that were not tested. The models were also used to extrapolate the data for new fatty acid structures (Scheme 1).

The ANN model appeared to be more accurate than the MLR model in predicting the mesophases of the system. The ANN model could effectively describe the complex phase behavior under different concentrations and temperatures. Comparison with the experimental data evidenced that the different predicted phases were interpolated with (high) accuracy ranging from 66% to 96%. The proposed approach allows to quantitatively study the influence of different factors on the formation process of different (meso) phases, thus elucidating the rules that are useful for future design and engineering of advanced nanoplatform. Finally, the machine learning approach may be useful in the prevision of the nanomaterials phase behavior, through the reduction of the number of experiments.

The gel-like LCs mesophases represent efficient non-toxic and biodegradable nanocarrier systems through different routes of administration as well as excellent nanoplatforms for controlled (and target selective) drug delivery processes [46,47]. For example, the successful incorporation of active compounds and drugs into LCNs has been exploited for the delivery of drugs through the blood–brain barrier (BBB), thus evidencing several advantages, like: Controlled drug release, improved colloidal stability (and drug bioavailability), reduced chemical and physiological degradation (in vivo) and reduction of side effects [47,48]. Moreover, entrapment of the curcumin into specific dispersion of monoolein-based liquid crystalline nanoparticle (with almost 100% encapsulation efficiency) evidenced enhancement of the colloidal stability of curcumin in the nano-formulation (about 75% of the curcumin is able to survive after 45 days of storage at T = 40 °C), while the in vitro release of curcumin was sustained (10% or less over 15 days) [49].

Despite the increasing interest in LCNs in several preclinical investigations that demonstrate the efficiency of their therapeutics release, different challenges need to be addressed before their translation into the clinic [46]. More specifically, a deeper investigation concerning their toxicity, intracellular trafficking and their connected interaction mechanism with cancer, cells and tissues is crucial for the development of novel biomedical applications of LCNs.

Finally, shape-memory and shape-changing photo-responsive liquid-crystalline polymer co-networks have been recently developed for the mimicking of various 2-D/3-D movements [50]. Despite the mechanical forces and the efficiency of photo energy conversion generated by these nanomaterials being quite low, this research field may stimulate future investigation oriented to the applications of the photo-responsive LCNs as sensors, actuators and muscles [50].

### 3.3. Self-assembly in Ternary Systems: Microemulsions

Microemulsions exist in optically transparent and thermodynamically stable amphiphilic systems formed by mixing two immiscible liquids (such as water and oil) that are stabilized by an appropriate surfactant (or combination of surfactant/cosurfactants) [51,52]. Depending on their composition, the following three typologies of microemulsions can be obtained, namely: the water in oil (W/O) microemulsions (wherein the water droplets are dispersed by the surfactants in a continuous oil phase), oil in water (O/W) microemulsion (wherein water droplets are dispersed in the oil the phase by the reverse micelles) and the bicontinuous microemulsions wherein nano-domains of oil and water are inter-dispersed within the system.

**Figure 6:**
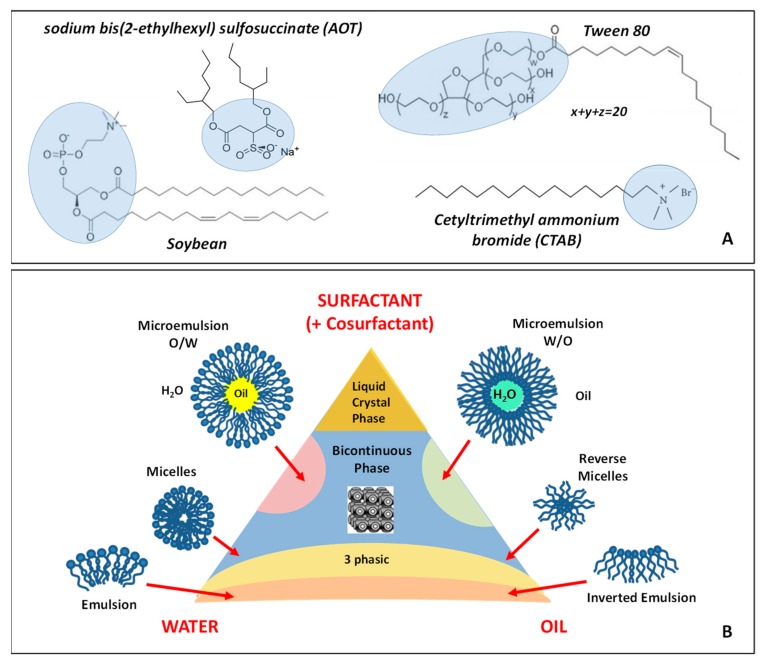
Chemical structure of some of the most important amphiphiles employed for the formation of microemulsions (**A**). Schematic representation of the possible different nanostructures that can be obtained by the self-assembly process in a system composed of water, oil and a surfactant (**B**).

A combination of several different types of ionic and non-ionic amphiphilic components can be employed to stabilize and increase the microemulsions colloidal stability region including the anionic sodium bis-2-ethylhexylsulphosuccinate (AOT), the cationic Cetyl trimethylammonium bromid (CTAB) and nonionic polyoxyethylene Brij 35 (C12E35) amphiphiles. Finally, the zwitterionic amphiphiles, such as the soybean and egg lecithin, are largely employed due to their excellent biocompatibility properties. In Figure 6A, we report the chemical structure of some of the most important amphiphiles employed for the formation of microemulsions.

The phase behavior of a microemulsion system can be characterized by several techniques, including transmission electron microscopy (TEM), scanning electron microscopy (SEM), cryo-electron microscopy (cryo-EM), interfacial tension, electrical conductivity, turbidity, rheology and (neutron and X-rays) scattering methods [53,54]. Figure 6B reports an example of a typical ternary phase diagram of water/oil/surfactant system. Microemulsions allow the solubilization of hydrophobic compounds in water-based systems and hydrophilic substances in oil-based systems. These properties not only allow the preparation and stabilization of clusters of exotic materials [55], but also can be exploited in a variety of applications in the nanomaterial areas including detergents, cosmetics, food and drug delivery, fuel, lubricants, corrosion inhibitors, coatings [56,57,58,59]. For example, either natural or synthetic (ionic/nonionic) surfactants, co-surfactants, and oils are used in cosmetic microemulsions such as skin/hair care products and perfumes [57,58]. More specifically, several bioactive compounds, that include skin whitening and antioxidants agents, have been delivered to the skin via cosmetic microemulsion products [57,58,59]. More specifically, the incorporation of glycolipids into the microemulsion in topical drug delivery applications, produced an increase of the skin permeation.

**Figure 7:**
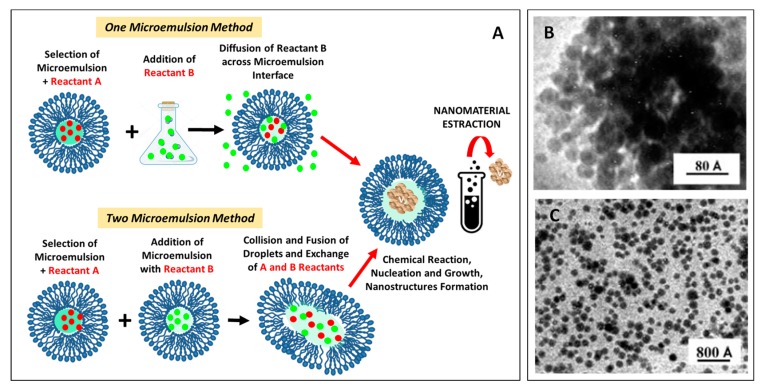
Sketch of the main approaches employed for the synthesis of nanoparticles with the One and Two Microemulsion methods (**A**). Transmission electron microscopy (TEM) image of the surfactant coated Na_2_S (**B**) and ZnSO_4_ (**C**) nanoparticles produced by using a Water/AOT/n-Heptane, W/O microemulsion [61].

Furthermore, the core region of the reverse micelle (reverse microemulsions) can play an active role as ‘chemical reactors’ for nanoparticles synthesis in the so-called microemulsion methods. [60]. In Figure 7A, we report a sketch of the main approaches employed for the synthesis of nanoparticles with the one (and two) microemulsion methods. This method is particularly fit for the preparation of water-insoluble materials. However, an interesting variation of this procedure has been set up for the preparation of Na_2_S and ZnSO_4_ nanoparticles by using a water/AOT/n-heptane microemulsions [61]. This approach is based on the total evaporation of the volatile components (water/apolar solvent) from a Water/AOT/n-Heptane W/O microemulsion, where a water soluble-substance has been previously dissolved. Due to the progressive elimination of the organic solvent, and thanks to the continuous inter-micellar exchange of materials, a progressive accumulation of the water-soluble substance within the polar hydrophilic cores of the micelles takes place [61]. The concomitant water extraction causes in-loco nucleation of the water-soluble substance in a process which has been defined “confined crystallization”. We report a TEM image of the corresponding surfactant coated Na_2_S (Figure 7B) and ZnSO_4_ (Figure 7C) nanoparticles produced by this approach [61].

Moreover, due to their high solubilization capacity and their ability to efficiently deliver both the hydrophobic and the hydrophilic drugs, they represent a powerful drug delivery system with improved drug solubility, penetrability (high diffusion/absorption rates), shelf life and ease of preparation [62,63]. Recent results indicate that microemulsions are predominantly employed in delivery of lipophilic drug (79.4%) by using oil-in-water microemulsions type. In this case, the oil phase is appropriately chosen to ensure and high drug solubility and an efficient drug transportation to the cells. For oral or topical administration, non-ionic surfactants are predominantly used (90%) [62]. Microemulsions that are employed for biomedical application consist of biocompatible and clinically safe components. Generally nonionic surfactants at low concentrations (for mild and non-invasive systems) are used, in order to lower the interfacial tension and to favour the mutual solubilization of the two (immiscible) liquids. Moreover, the cancer treatment is the most targeted disease in drug delivery applications, followed by the inflammatory diseases, microbial (infections) and cardiovascular disease [61,62]. Recently, O/W microemulsions composed of Tween 80 surfactant and triacetin as dispersed oil phase were investigated as delivery vehicles of the Vemurafenib analog PLX4720 an antitumor (lipophilic) drug against various types of cancer [63]. In this case, the cell viability assays on the colon cancer cell lines (Colo-205 and HT29) showed no cytotoxicity effects when the microemulsions were added in the ratios between 0.005% v/v and 0.2% v/v [63].

## 4. Polymer-based Building Blocks: Linear, Cross-linked and Hyperbranched/Dendritic Morphologies

Other important classes of organic compounds for self-assembly processes are represented by polymer-based (macromolecular) building blocks. The advancement of polymer science and technology allows a versatile synthesis and modification of polymers chemical groups. Polymer processing nanotechnology has been employed for the conjugation and functionalization of active compounds of many polymer-based nanostructured materials [64]. Depending on their molecular topology and their binding sites, the polymer building blocks can be classified into the following three main topologies:

- ***Linear polymers***
- ***Cross-linked polymers (nano-gels)***
- ***Hyperbranched/dendritic polymers***

### 4.1. Linear Polymers

#### 4.1.1. Homopolymers Building Blocks: Polymer-based Nanoparticles

The development of polymer-based nanostructures and nanoparticles by means of the self-assembly approach is assuming a key role in various fields of the nanotechnology area, including photonics, sensors, electronics, biomaterials, nanomedicine and environmental technology [64,65]. Moreover, the development of polymer-based nanoparticles (PNPs) by self-assembly processes allows the formation of functional nanoplatform for advanced biotechnology application in the area of optical diagnosis including biomarker screening, cancer and immunoassays diagnosis [66]. For the detection of the target analyte, the binding of the target biomolecule with PNPs should produce a measurable (quantified) signal. Moreover, many biodegradable polymers demonstrated promising performances in biomedical and drug delivery applications. More specifically, they provide a high control over the structure-function relationship and a controlled drug release by crossing the physiological/pathological barriers of living systems [66,67].

In the past years, a wide range of natural polymers has been studied for drug delivery purposes including dextran, chitosan, hyaluronan and heparin [64,67,68,69]. Amongst all the commonly used biodegradable synthetic polymeric (bio-)materials the poly-(ε-caprolactone) (PCL) [70], poly(lactic acid) (PLA) [71] and poly(D, L-lactic-co-glycolic acid) (PLGA) [72] are the most commonly employed polymers for drug delivery applications. Due to their biocompatibility, low levels of toxicity and immunogenicity and their easily (tunable) biodegradability in vivo, these classes of polymers have been approved by U.S. Food and Drug Administration (FDA) and the European Medicines Agency (EMA) as effective drug delivery carriers in humans. Depending on the self-assembly strategy the chemical composition and nature of the attached ligand, various typologies of PNPs with different dimensions, shapes, and physicochemical properties can be tailored for various biopharmaceutical and imaging applications [73,74].

**Figure 8:**
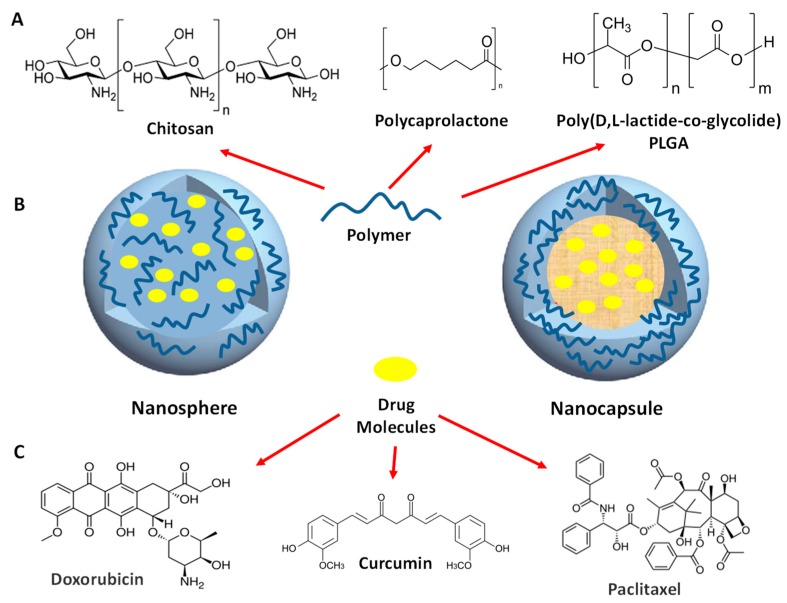
Example of some of the main polymer building blocks (**A**) employed for the formation of the polymeric nanocapsule and nanosphere (**B**). We report some of the main active drug macromolecules employed in biomedical applications of polymer nanoparticles (Doxorubicin, Curcumin, Paclixatel) (**C**).

Research on therapeutic PNPs has been focused on anti-inflammatory, antineoplastic, immunosuppressant, hormone, antigen, antibacterial, antiviral, diuretic and antifungal applications [73,74]. From the structural point of view, PNPs can be divided into two main families: the nanospheres, which exhibit a homogeneous structure across the nanoparticle, and the nanocapsules, which have a typical core-shell internal structure that provides protection of the active molecules from the degradation performed by external factors (such as the enzymatic attack during the digestive process). We report some of the main polymer building blocks (Figure 8A) employed for the formation of polymeric nanocapsule and nanosphere (Figure 8B), together with some of the main active drug macromolecules employed in biomedical applications (Figure 8C).

#### 4.1.2. Amphiphilic Block Copolymers

Self-assembly of amphiphilic block copolymers represents a versatile tool for the design and engineering of advanced materials, as well as an important approach to investigate the complex processes in the field of soft matter and colloidal science [12,75,76,77,78]. These systems offer the possibility of molecular control by tuning the architecture and polymer composition. Self-assembly of linear amphiphilic block copolymers in selective solvents can be viewed in strong analogy with the self-assembly of surfactant molecules. Moreover, the large number (>500) of monomeric units may favor a more efficient polymer entanglement and a stronger inter-block interaction in comparison with traditional low molecular weight surfactants. Together with the linear AB diblock copolymers, thermoresponsive linear ABA (or BAB) triblock architectures have attracted enormous scientific interest and have already found broad application in material science, biotechnology and nanomedicine [79,80,81]. Furthermore, the use of polymeric amphiphiles represents a promising bottom-up method for the self-assembly processes of organic-inorganic porous materials, in which an (macromolecular) amphiphilic template drives the self-assembly of nanostructures with desired final properties (soft-templating approach) [7,82,83].

The combination of block copolymers’ interactions together and the efficient control of the assembly’s structural morphologies make amphiphilic block copolymers excellent templates in the construction of nanostructured porous materials with advanced properties [7,82,83]. Soft-templated mesoporous materials have attracted the interests of researchers in a large variety of fields including catalysis, adsorption and separation processes, optics, electronics, host-guest chemistry and bio-nanotechnology [7,82,83].

**Figure 9:**
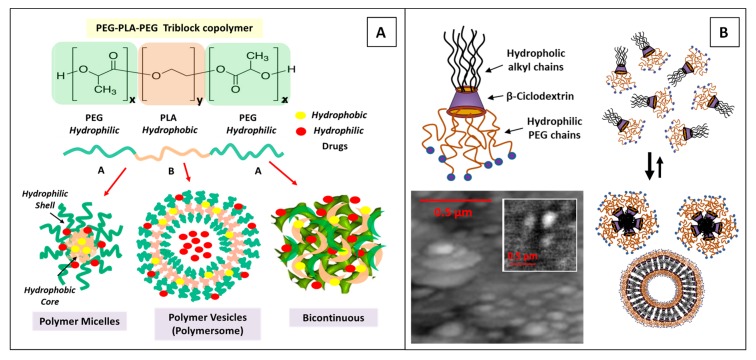
Structural features of the amphiphilic poly(ethylene glycol)-polylactide-poly(ethylene glycol) (PEG-PLA-PEG) triblock copolymers and its use as drug delivery nanocarrier (in the form of micelles, vesicles and bicontinuous phase) for both hydrophobic and hydrophilic drugs (**A**). Self-assembly process of cationic SC6CDNH2 modified cyclodextrins (**B**), and the relative SNOM optical images (inset) of the aggregates obtained by the self-assembly process between anionic TPPS porphyrins and cationic modified amphiphilic cyclodextrins (at 1:10 porphyrin/CD molar ratio).

Moreover, self-assembly of amphiphilic block copolymers represents a versatile tool for the design of versatile nanocarriers for drug delivery applications. More specifically, the di- and tri-block copolymer of poly(ethylene glycol)/polylactide (PEG/PLA) systems, in the form of both polymer micelles and vesicles (polymersome), are attractive delivery systems since they are biodegradable with a good safety profile and sustained drug delivery [84,85]. The presence of the PEG on the nanocarrier shell prevents the unwanted adsorption of proteins and phagocytes, thereby increasing the period of the blood circulation, while the PLA hydrophobic core can efficiently encapsulate a variety of therapeutic agents [12,80]. Block copolymers micelles, polymersomes and bicontinuous phases exhibit an enhanced efficiency in solubilizing relevant doses of hydrophobic and hydrophilic drugs (Figure 9A) [85,86].

Linear Pluronic-type ABA amphiphilic triblock copolymers of poly(ethyleneoxide)-poly(propyleneoxide)-poly(ethyleneoxide) (PEOm-PPOn-PEOm), also known as polaxamers, represent another interesting polymer system for versatile applications in the biotechnology and nanomedicine fields [86]. The rich variety of aggregation phases (micelles, polymersomes, lyotropic liquid crystals, hydrogels, etc.) of pluronic block copolymers makes them suitable to a multiple typology of processing routes and various nanomedicine applications [86]. Due to their encapsulation properties, that exhibit increased solubilization efficiency (in the PPO core region) and efficient steric stabilization (from PEO corona), and their ability to undergo specific sol-to-gel phase transitions upon heating, they have shown promising results as smart nanocarriers in the delivery of anticancer drugs [86,87]. Targeted drug delivery can improve the efficacy and reduction of side effects by designing more selective, long-circulating and stimuli-responsive nanoformulations [81,86]. Furthermore, PEO-PPO-PEO block copolymers have shown promising results in both non-viral and viral gene transfer applications and as advanced gene delivery nanoplatform in various regenerative nanomedicine applications [87].

Recently, amphiphilic hyperbranched multiarm copolymers (HMCs) have displayed great potential as excellent nanomaterials building blocks [88,89,90,91,92]. The highly branched polymer-based amphiphiles present interesting properties resulting from the (hyper-) branched structure that generate nanostructures with high solubility, high colloidal stability.

Many interesting supramolecular nanoaggregates and hybrid nanostructures, such as tubes, fibres, vesicles, micelles and honeycomb films have been generated [89,90,91,92]. Mazzaglia et al. developed a novel heterotopic nanocarrier formed between the anionic porphyrin 5,10,15,20-tetrakis(4-sulfonatophenyl)-21H,23H-porphine (TPPS) and a cationic modified (amphiphilic) cyclodextrins (SC6CDNH2) (Figure 9B), for the photodynamic therapy of tumors [90]. They evidenced that the electrostatic interaction between anionic TPPS porphyrins and the cationic SC6CDNH2 modified cyclodextrins evidenced the important role of nanocarrier charges in the control of the colloidal stability of the nanocarrier. We report the self-assembly process of cationic SC6CDNH2 modified cyclodextrins (Figure 9B), and the the topography and relative optical SNOM images (inset) of the aggregates obtained by the self-assembly process between anionic TPPS porphyrins and cationic modified amphiphilic cyclodextrins (at 1:10 porphyrin/CD molar ratio) [90].

Finally, the HMCs systems have received great interest in both academic research and industrial fields because of their excellent properties for a broad range of nanoscience and nanotechnology applications, including energy harvesting, catalysis, photonics, drug delivery and nanomedicine applications [89,90,91,92].

**Scheme 2:**
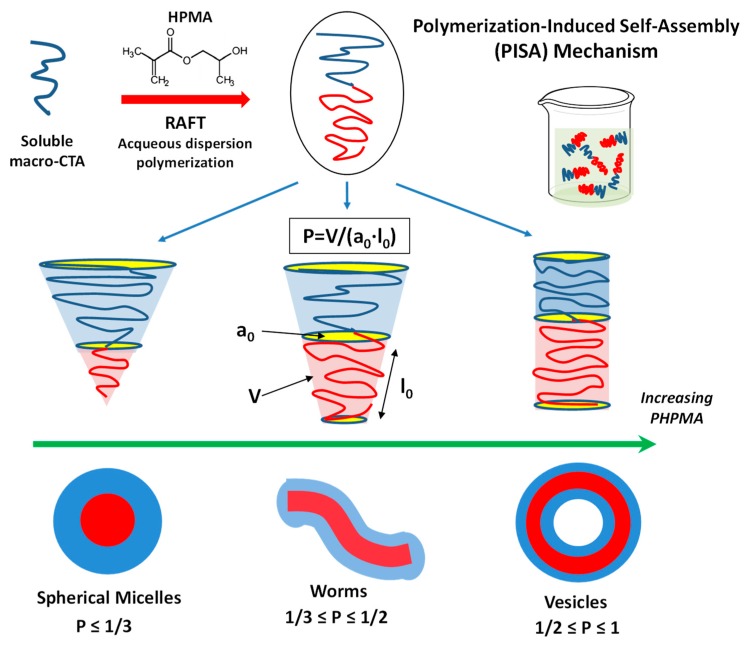
(Top) Schematic representation of the polymerization-induced self-assembly (PISA) approach. A soluble macromolecular chain transfer agent (macro-CTA) (blue) is chain-extended by an initially miscible monomer to yield the growth of an insoluble polymer block (red). (Bottom) Depending on the degree of polymerization of 2-hydroxypropyl methacrylate (HPMA), the amphiphilic block copolymers self-assemble in a variety of final morphologies, which depend on the geometrical and conformational factors, through the packing parameter *p*.

Over the past decades, particular interest has been paid on an alternative route for the formation of block copolymer nano-assemblies known as *polymerization-induced self-assembly (PISA)* [93]. The PISA approach consists on the self-assembly of polymer chains that is performed at the same time with the polymerization process [93]. The PISA formation process is shown schematically in Scheme 2.

Typically, a soluble homopolymer (A), consisting in a macromolecular chain transfer agent (macro-CTA), is chain-extended using a second monomer (B) (in a suitable solvent). Successively, the growing second block (B) becomes gradually insoluble and drives the (in situ) self-assembly to form AB diblock copolymer nano-objects. The A block, that acts as a steric stabilizer, is usually prepared via solution polymerization, while the insoluble B block is prepared via either dispersion or aqueous emulsion polymerization. A variety of macro-CTAs may be used to generate various nano-assemblies, such as the 2-hydroxypropyl methacrylate (HPMA). By varying the degree of polymerization (DPs) of the two blocks, a large variety of morphology can be obtained, including spheres (micelles), worms or vesicles.

Although the PISA method can be performed by means of any type of living polymerization, in the majority of cases the reversible addition−fragmentation chain transfer (RAFT) polymerization is used. This approach allows the environmentally friendly production (in large scale) of nano-assemblies, in relatively high concentrated solution (25−50% w/w) and with easily tunable sizes and shapes [93]. Various parameters allow the control of the morphology of PISA nanostructures, including the choice of core-forming polymers (and end-groups) and the solution pH. Finally, the choice of specific macro-CTA, together with its degree of polymerization, concentration and its thermosresponsitivity, represents crucial control parameters of the PISA approach [93]. The main limitation and challenges of the PISA technique is connected with the uncertainty to predict the morphology of the nanoparticles without a correlated investigation of the phase diagram.

Recently, the PISA approach has been employed for the synthesis of novel fluorescent nanoparticles (obtained by labelling the nanoparticles core with a fluorescent dye) and their interaction with (primary) human umbilical vein endothelial cells (HUVECs) [94]. This approach allows an improved investigation of the nanostructure’s interactions under flow conditions, thus allowing the employment of blood to mimic the human vascular network [94].

Different nanostructures morphologies of ^19^F-containing polymeric nanoparticles, including vesicles, spheres and worm-like nanostructures, were recently synthetized by means of the PISA approach for cell uptake and tracking applications [95]. This investigation evidenced that the cell uptake process depends on the morphology of the nano-objects, with a higher uptake action for worm-like particles in comparison to spherical and vesicles nanoparticles [95].

Furthermore, a library of nanoparticles were prepared by the PISA approach combining a polystyrene core with a PEG corona composed of poly(glycidyl methacrylate)-b-poly(oligo(ethylene glycol) methyl ether methacrylate) [96]. By varying the hydrophobic polystyrene block length, the authors were able to control the size and morphology of the nanoparticles without affecting the PEG corona surface chemistry. The study of the biodistribution, carried out in nude mice bearing HT1080 tumor xenografts (48 h after intravenous administration), evidenced that the smaller spherical micelles (diameter 21 nm) exhibited the highest tumor accumulation compared with the larger spherical (diameter 33 nm), rodlike (diameter 37 nm, contour length 350–500 nm) or wormlike (diameter 45 nm, contour length 1–2 μm) micelles [96].

**Figure 10:**
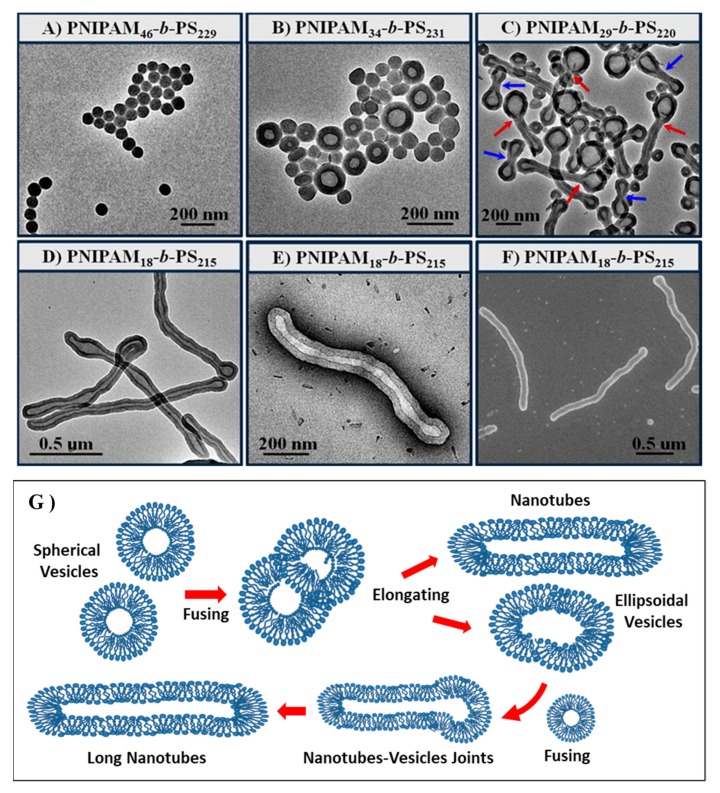
TEM (**A**–**E**) and scanning electron microscopy (SEM) (**F**) images of the various PNIPAM-b-PS diblock copolymers nanoaggregates prepared via PEG-PISA (in PEG400). Formation mechanism proposed for the nanotubes and ellipsoidal vesicles under the PISA approach in Poly(ethylene glycol) (**G**). Adapted with permission from ref. [97]. Copyright (2016) American Chemical Society.

Recently, a PISA formulation via dispersion RAFT polymerization in poly(ethylene glycol) (PEG) was proposed for the synthesis of a large variety of amphiphilic diblock copolymer systems, including poly(di(ethylene glycol) methyl ether methacrylate)-block-polystyrene (PDEGMA-b-PS), poly((ethylene glycol) monomethyl ether)-block-poly(tert-butyl acrylate) (mPEG-b-PtBA), poly(N,N-dimethylacrylamide)-block-polystyrene (PDMA-b-PS), poly(4-vinylpyridine)-block-polystyrene (P4VP-b-PS), poly(N-isopropylacrylamide)-block-polystyrene (PNIPAM-b-PS), and the doubly hydrophobic PMMA-b-PS diblock copolymer [97]. This PEG-PISA approach provides a good control over the (fast) in situ synthesis of highly concentrated (20–50 wt %) diblock copolymer. Together with the vesicles, nanospheres, worms and lamellaes aggregates, two new morphologies of nanotubes and ellipsoidal vesicles have been prepared from the PNIPAM-b-PS diblock copolymer system as evidenced in by the images of the scanning electron microscopy (SEM) (Figure 10A–E)) and transmission electron microscopy (TEM) (Figure 10F). Figure 10G reports the corresponding formation mechanism proposed for the nanotubes and ellipsoidal vesicles aggregates under PEG-PISA approach [97].

Finally, a critical issue in the self-assembly processes involving the block copolymer is represented by morphology instability induced in the nano-assemblies by the presence of given amounts of surfactants. In this respect, the various morphologies (such as worm, vesicle and lamellar structures) typically generated by these synthesis techniques are not able to tolerate the presence of added surfactants, thus limiting their potential applications.

In a recent study, the enhancement of the colloidal stability (on the various morphologies of non-crosslinked nano-assemblies) in concentrated solutions of sodium dodecyl sulfate (SDS) surfactant was obtained by means of the RAFT emulsion polymerization, by using poly(di(ethylene glycol) ethyl ether methacrylate-co-N-(2-hydroxypropyl) methacrylamide) P(DEGMA29-co-HPMA6) as a macro-CTA [98]. In this investigation, the surfactant tolerance was caused by the limited binding of the SDS surfactant to the main-chain of the P(DEGMA-co-HPMA) macro-CTA (constituting the nanoparticle shell). This investigation may stimulate novel application of non-crosslinked nanostructures formed by means of RAFT emulsion polymerization techniques for the preparation surfactant-tolerant nanomaterials [98].

### 4.2. Cross-linked Polymers (Nanogels)

Polymer co-networks (nanogels and hydrogels) consist of cross-linked polymeric networks that contain both hydrophilic (or polar) and hydrophobic monomer units, that are able to swell their volume in response to (internal) environment changes or external stimuli [99,100,101]. The hydrogel properties can be modified to match the requirements of specific applications by selecting the polymers type (and length), the cross-linking method. Moreover, the presence of basic (or acidic) end-groups, allows to modify the protonation state by controlling the pH or salt concentration.

**Figure 11:**
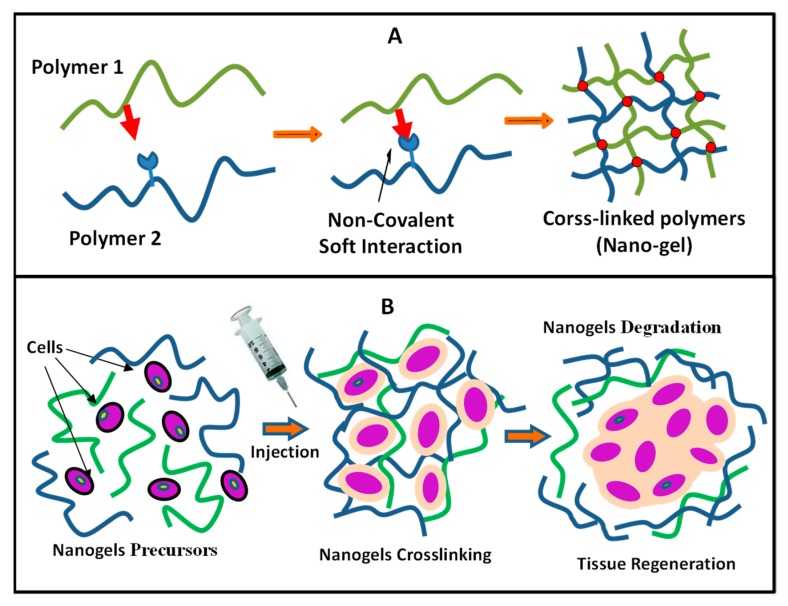
Schematic representation of the non-covalent forces acting on a cross-linked polymer (nano-gel) (**A**). In situ formation of a scaffold in tissue engineering application by injection into the wound of a polymer/cell solution and formation of a cross-linked polymer network (**B**).

Moreover, some stimuli-responsive nanogels are able to perform mechanical work or change its aggregation state in response to changes of external physical properties (stimuli), such as the light or electric field exposure, and its temperature [101]. The hydrosoluble Poly(N-isopropylacrylamide) (PNIPAM), Chitosan, Poy(vinil alcohol), alginate hydrogels are among the most intensely investigated polymer systems for advanced biomedical applications [101,102]. The main applications of stimuli-responsive hydrogels include microactuators and microfluidic devices, electrolyte batteries, artificial muscles and superadsorbents [100,101,102]. The pH-responsive gels have been mainly employed for hydrophilic drug delivery. In this case, the drug release occurs by diffusion within the swollen polymeric network. The pH-responsive gels are particularly versatile for the targeted drug delivery. In biomedical research, the responsive hydrogels have been found to have applications in the fields of drug delivery, biosensors and bio-mimetic nanomaterials [101,102]. Moreover, hydrogels and nano-gels have been used as versatile tissue engineering scaffolds due to their ability to restore, retain and regenerate the damaged tissues. They are able to provide an excellent environment for cell survival, with a molecular structure which is able to mimic the bio-tissues or serving as a functional matrix for efficient cell immobilization and active compounds delivery [95,96]. In Figure 10, we report the main self-assembly mechanism for the structural formation of a cross-linked nanogel (Figure 11A) and the main mechanism of the (in situ) formation process of a scaffold for tissue engineering application (Figure 11B).

Recently, a new design approach was proposed to increase the mechanical properties of agar/poly(acrylic acid)/hydroxyethyl cellulose (composite) hydrogels prepared by a one-pot method [103]. The insertion of the polyhydroxy compounds allows the formation of a (physically linked) double network (DN), by exploiting the inter-polymers hydrogen bonding interaction [103]. The resulting hydrogels evidenced optimal performances for potential application in biomedical fields [103]. Furthermore, a chitosan-graft-poly(ε-caprolactone) (CS-g-PCL) copolymer was studied (in hydrated and dry state) [104] and its performances was compared to those of the individual homopolymers, poly(ε-caprolactone) (PCL) and chitosan (CS) [104]. The in vitro mechanical and biological evaluation evidenced that the CS-g-PCL copolymer is able to support the growth of Wharton’s jelly mesenchymal stem cells, the tissue formation and a simultaneous nanomaterial degradation, thus evidencing possible application as a scaffold in soft-tissue engineering [104].

### 4.3. Hyperbranched/Dendritic Polymers: Self-assembly and Co-assembly of Dendrimers

Highly branched polymeric systems (such as dendrimers and hyperbranched polymers) possess many special properties, such as a high number of functional endgroups, high densities and high colloidal stability in solution. These properties make such polymer systems special candidates for a large variety of nanomaterial applications, including template scaffolds for nanostructure synthesis, targeted drug delivery, nano-support for catalysts [105]. Within this class of highly branched polymeric systems, a special role is played by dendrimers.

**Figure 12:**
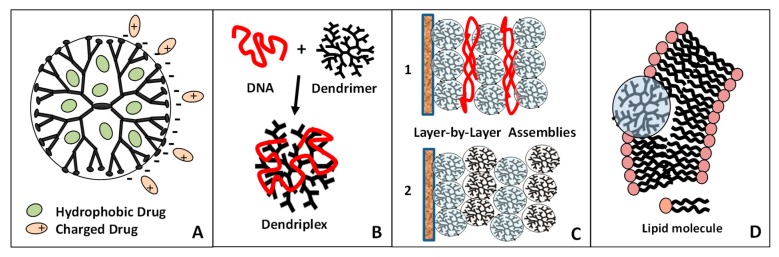
Inclusion and assembly of hydrophobic and hydrophilic drugs within the dendrimers structure (**A**). DNA polyelectrolyte and dendrimer interaction and formation of a dendrimer/DNA complex (called Dendriplex) (**B**). Detection support formed by dendrimer layer-by-layer (LbL) nano-assemblies (**C**). Interaction between dendrimers and lipid membrane and self-assembly of dendrimer-lipids nanostructured complexes (**D**).

Dendrimers are highly branched nanostructures, consisting of polymeric branching segments organized in concentric layers (generations) attached to a central core, and terminating with a given number of surface functional endgroups [106,107]. Self-assembly processes between dendrimers and inclusion components allow the development of novel technologies in the field of material science and biotechnology [108]. Hydrophobic active compounds (such as drugs) may be entrapped within their internal intramolecular cavity, while the charged species may interact with the dendrimers charged moieties and can be conjugated to their functional groups in the surface (see Figure 12A) [109,110].

The study of the electrostatic interaction of charged dendrimes is particularly interesting, as it can be exploited for the self-assembly processes in various complex nanostructure formation of biotechnological interest [110,111,112]. For example, when DNA is mixed with amine terminated polyamidoamine (PAMAM) dendrimers, it favors the formation of a complex (called Dendriplex) that presents a more compact structure, due to the electrostatic interactions between the (cationic) PAMAM dendrimers and the (anionic) DNA polyelectrolyte (Figure 12B). Several investigations have evidenced that the solution properties (including ionic strength and pH) play a fundamental role in the control of the dendrimer’s electrostatic interactions. Electrostatic and other non-covalent soft interactions can be exploited for the development of a variety of functional nanostructures suitable for advanced nanotechnology applications [113,114,115,116,117].

Moreover, self-assembly involving dendrimer building blocks is employed for the construction of biodetection systems (biosensors) by using electrodes (or optical transducers) coupled with molecular recognition components (such as enzymes or antibodies) [118,119]. In this case detection technologies are based on the immobilization on a support formed by dendrimer layer-by-layer (LbL) assemblies [118,119]. The LbL nanostructured are assembled by using positively charged dendrimers in combination with polyanions (or negatively charged dendrimers combined with polycations) (Figure 12C-1). Finally, LbL films may be built starting from the self-assembly of oppositely charged dendrimers (Figure 12C-2) [118,119].

Another interesting self-assembly process involving dendrimers nanoparticles consists in the formation of the dendrimer-lipids complexes. The different mechanisms of the (dendrimers–lipids) interactions (including hole formation, adsorption on membrane, and vesicles disruption) [120,121,122,123,124], strongly depend on the force balance between the surface charge of the dendrimers and the (zwitterionic/charged) lipids headgroups, and on the hydrophobic interaction between dendrimers and the lipids hydrocarbon chains [122,123,124]. These processes have important implications for the comprehension of mechanism of translocation of dendrimers in bio-membranes and living cells.

Furthermore, dendrimers are promising nano-carriers of genes therapy [125,126,127,128]. Usually, the positive charge in the surface of most dendrimers favors the formation of complexes with nucleic acids (dendriplexes) that maintain a positive net charge, thus facilitating the binding with the cell membranes. In this case, the dendriplexes undergo the cellular (endosome) uptake followed by the internalization inside the cytoplasm. These processes are followed by the destruction of the dendriplex and the successive release of the nucleic acid within the cytoplasm. Finally, the DNA enters the nucleus for successive gene expression [125,126,127]. It has been found that the transfection efficiency, which is mediated by PAMAM dendrimers, depends on the charge ratio of the complexes and on the dendrimer size (with larger generation dendrimers providing the higher efficiency).

Furthermore, the use of dendrimers building blocks allows to increase both the pharmaco-kinetics and pharmaco-dynamic properties of the assembled nanocarriers, thus stimulating many therapeutic investigations including diabetic, Parkinson’s disease, glaucoma, anticancer and antimicrobial agents [128,129]. Finally, coordination chemistry has been used to prepare a great variety of dendrimers-based supramolecular nanomaterials and dendritic hybrids nanoplatforms [130,131]. With appropriate choices of dendrimers containing both metal centers (or nanocrystals) and complementary functional moieties, it is possible to control dimension, morphology, functional properties and stability of most accessible and programmable building blocks, such as the minidendrimers, minidendrons and dendronized polymers [130,131]. The development of the so called “lock and key” assembling processes stimulated different investigations in areas of supramolecular dendrimers assemblies including signaling, diagnostics and energy harvesting and conversion [130,131].

## 5. Self-assembly by Biomolecules Building Blocks

The field of the biomolecular self-assembly is an emerging field at the intersection of various disciplines including physical and chemical sciences, molecular biology, materials science and engineering. A variety of biomolecules including peptides, proteins (viruses and enzymes), lipids, and oligonucleotides (DNA/RNA) macromolecules exhibited great potential to form supramolecular functional nanostructures. Due to their excellent physico-chemical properties and their higher reactivity than conventional non-biological building blocks, these biomolecules allow to develop a large variety of nanostructures, characterized by shape accuracy architectures that can be employed for the design and engineering of advanced nanodevices in the fields of materials science, biotechnology and biomedical engineering [132,133,134].

### 5.1. Peptide and Protein Based Bio-Nanomaterials

**Figure 13:**
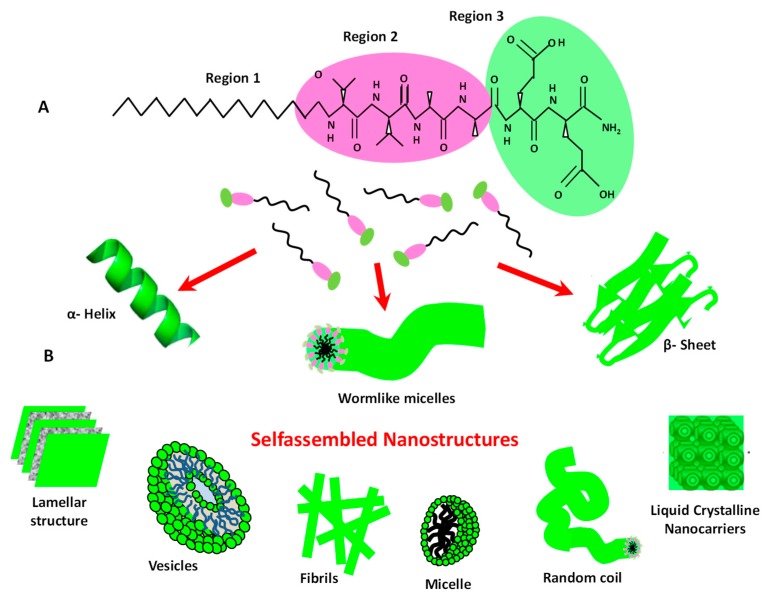
Typical (3 regions) structure of a peptide amphiphile (**A**). Example of the possible nanostructures generated by the peptide amphiphiles self-assembly (**B**).

Peptides serve as versatile building blocks for bio- and nanotechnology applications owing to the ease of chemical and biological modifiability by synthesis and their high bio-reactivity. The study of the self-assembly properties of peptides has been exploited in the field of biotechnology and nanomedicine for the formation of bioinspired (biocompatible and biodegradable) nanostructures, including nanospheres, nanorods, nanotubes, nanofibers and hydrogels (Figure 13) [134,135]. The self-assembly processes of polypeptides into amyloid fibrils nanoaggregates is connected with specific diseases such as the human Alzheimer’s disease, Crutzfeld–Jakob disease, type II diabetes and various microbial physiological processes [136].

The peptides self-assembly, which is controlled by the amino acid (length and typology) sequence, strongly depends on the subtle combination of non-covalent interactions including hydrogen bonding, hydrophobic and electrostatic interactions, van der Waals forces and π–π stacking [135]. In aqueous solution the peptides hydrophobic residues shield themselves from the water while the hydrophilic endgroups are exposed at the external surface. Peptide self-assembly is also driven by the folding process, whereby the folded surfaces self-assemble into nano-domains with different secondary structures, including α helix, β sheet (and β turn). Moreover, the peptide self-assembly can be tuned by controlling the concentration, temperature, pH and other parameters, with the scope to developed biomaterials which are responsive to multiple (external/environmental) stimuli [135,136]. The engineering of self-assembling peptides involves the choice of short peptide sequences in order to drive the formation of well-defined secondary structures (like α-helices or β-sheets), and their conjugation to the hydrophobic chains.

The special class of peptide amphiphiles (PAs) are composed of hydrophobic chains conjugated to hydrophilic bioactive segments, that can be properly modified to obtain the programmed functionality, such as stimuli-responsiveness, targeting and cell-penetrating functions [137]. As shown in Figure 13A, they are generally composed of three regions: (region 1) formed by an hydrophobic aliphatic chain that modulate, with their length, the system hydrophobicity; (region 2) existing in a defined peptide sequence with β-sheet propensity and rich in hydrophobic acids, (such as leucine, valine and alanine); and (region 3) composed of a bioactive head that can be further functionalized with bioactive components [137]. In aqueous solution, the PAs self-assemble into a large range of novel nanostructures (Figure 12B) including elongated micelles, bilayer vesicles, lamellar sheets, fibrils and LCNs (Figure 12).

Self-assembly processes involving PAs is the subject of intense research investigation, exploring the role of charged moieties, the influence of the amino acid sequences, and the activity of their specific functional groups. Potential applications of PAs include drug delivery, tissue engineering and regenerative medicine [135,136,137,138,139]. Short chain cationic peptides may exhibit antimicrobial properties, which are enhanced by lipidation, which causes some morphologic changes in secondary structure during the peptide interaction with the bio-membranes [138]. Recently, the simple mixing of PAs nanofibers with a (chemically) cross-linked poly(ethylene glycol) (PEG) polymer network undergoing a photopolymerization, resulted in the formation of a porous composite hydrogel that can be functionalized with desired bioactive signaling epitopes by altering the PAs amino acid sequence [139]. The nanofibrous self-assembled PA/PEG composite hydrogels provide a versatile technique to create synthetic extracellular matrix (ECM) analogues with tunable bio-mechanical properties for regenerative medicine and tissue engineering applications [139].

There are also great research efforts to synthetize novel peptide-mimetics nanostructures based on peptoids and their conjugates. Cell penetrating peptides (CPPs) are a specific short sequence of peptides that facilitate the cellular uptake of various macromolecules [140]. They are able to enter cells with an energy-independent manner by means of interactions that trigger some specific endocytic pathways [140]. A variety of CPPs has been synthesized and utilized to translocate a variety of active compounds, such nucleic acids, proteins, nanoparticles and imaging and contrast agents in preclinical (and clinical) investigations [141]. Recently, CPPs were utilized in the enhancement of the intracellular delivery efficiency of viral nanoparticles, which caused a reduction of the consumption of materials, thus allowing a minimization of virus nanoparticles cytotoxicity, and reducing the risks in their potential clinical application [142].

Finally, proteins represent an excellent tool in modern nanotechnology, whereby their tertiary structures serves as building blocks for the formation of quaternary structures and self-assembled functional nanostructures [143,144]. Protein building blocks can be functionalized via chemical or biological strategies for the development of protein supramolecular nanostructures with different morphologies including strings, tubules, nanorod, layers, cages, vesicles, nanocrystals and nanogels, etc. [137]. The unique proteins structure of enzymes and antibodies permits only the attachment to other unique structures, thus allowing a controlled self-assembly process by joining together two or more specific macromolecular building blocks (molecular recognition). The protein self-assembly approaches utilize a number of specific interactions including covalent reactions, hydrogen bond, Van der Waals and electrostatic interactions, receptor–ligand recognitions and metal–ligand coordination. They have been employed for the formation of hierarchical protein nanostructures including, 1D (strings/tubules/nanowires), 2D two-dimensional (nanorings/networks) and 3D (hydrogels and crystalline frameworks) functional nano-assemblies [145]. Their advanced biological functions allow various nanotechnology applications including biomimetic protocells, artificial enzyme, protein-based hydrogel, as well as photosynthesis, drug carriers and imaging nanoplatform [144,145]. Understanding the physico-chemical factors that regulate the peptide and protein self-assembly processes represent the first crucial step for the design and engineering of new building blocks for advanced biotechnology and nanomedicine applications.

### 5.2. Lipids

Lipids are excellent building blocks for advanced nanotechnology applications, due to their ability to self-assemble into a large variety of morphologies including nano-films, nanorods, liquid crystal, bicontinuous nanostructures, (reverse) micelles and liposomes [146]. Natural or synthetic lipids are more biocompatible than the inorganic and polymeric nanomaterials and present a marked ability to interact with biological tissues and biomembranes. More specifically, phospholipids, the most abundant lipids components in biomembranes of living organisms, are composed of amphiphilic molecules with neutral or (positively/negatively) charged hydrophilic headgroups linked to (one or more) hydrophobic tails [147].

When dissolved in aqueous solutions, lipids molecules generally self-assemble to form (metastable) bilayer morphologies such as lamellar or vesicles nanostructures [146,147,148]. Depending on the preparation methods (i.e., sonication, extrusion, stirring), vesicles nanostructures (with sizes ranging between 10 nm and 10 μm) may have one (unilamellar) or more (multilamellar) onion-like concentric bilayers (hydrated multilayers) [148,149].

The formation of lipid nanostructured systems is controlled by soft interactions that regulate their colloidal stability in harsh bio-environments thus allowing a better control over their nanostructure [149,150,151,152]. For these reasons, lipid nanostructures are considered a versatile nano-platform in the field of nanomedicine and biotechnology [153,154]. Recently, a P3 lipopolymer (comprising of prostate-specific membrane antigen (PSMA) ligand, polyethylene glycol (PEG2000), and palmitate) was employed for the functionalization of liposomes for the targeted delivery of drugs and diagnostics to PSMA+ advanced/metastatic prostate tumors [155]. The investigation showed that doxorubicin loaded functionalized liposomes evidenced a higher toxicity to human prostate cancer LNCaP cells lines with respect to the non-targeted liposomes. Furthermore, the inclusion of liposomes in thermosensitive hydrogels directly affected the hydrogel capabilities and favored the viability as well as the differentiation of PC12 cells [156]. This effect, which depends on the lipid type, allows the development of novel biomaterials for neural tissue engineering application. Recently, hydrogels with different shapes (e.g., cube, sphere, hexagon) were coated with lipid monolayer and employed as efficient building blocks for the construction of (scalable) nano-microelectronic devices and as nano-micromechanical systems, such as switches, rotors (driven by magnetic field) and painted circuits [157].

*Solid lipid nanoparticles (SLNs)* are small (50–200 nm) nanoparticles with a solid lipid matrix (at the room temperature). They consist of a mixture of various lipids and small concentration (about 1–5% w/v) of surfactants or cosurfactants that stabilize the core region [158,159]. SLNs are able to naturally cross the blood-brain barrier (BBB) due to their highly lipophilic nature (passive targeting). Brain uptake is performed by the paracellular pathway through the opening of the tight junctions in the brain microvasculature. Brain targeting with apolipoprotein-E receptors, which are predominantly expressed in the brain, facilitated transport across the BBB (active targeting) [158].

*Nanostructured lipid carriers (NLCs)*, a slightly modified version of SLNs, consists of a mixture of liquid/solid lipids and the addition of mono-, di- and triglycerides lipids (with different chain lengths) [158,160]. This composition causes imperfections in the crystal structure, thus increasing the internal free space of the solid, and resulting in a higher drug loading efficiency. Both SLNs and NLCs are employed in a large variety of applications, including oral and pulmonary drug delivery, food, cosmetic, gene transfer [158,159,160,161].

*Bilayer vesicles* (called liposomes), that are composed of (natural or synthetic) lipids, are excellent drug delivery nano-platforms, due to their versatile self-assembly process, which is governed by a variety of soft interactions that allow a control of their structures and enhancement of their colloidal stability in the complex bio-environments of diseased tissues [162,163]. Liposomes have unique properties, as their size, charge, and surface properties that can be easily tuned by selecting different components of the lipid mixture ingredients. While hydrophobic drugs are localized within the hydrophobic alkyl chains of the liposome, the hydrophilic drugs are hosted near the hydrophilic headgroups region or in the internal aqueous core region. As many therapeutic drugs present an intermediate solubility, they undergo a partition between the external (or internal) aqueous phase of the liposome and the hydrophobic internal region of the bilayer.

**Figure 14:**
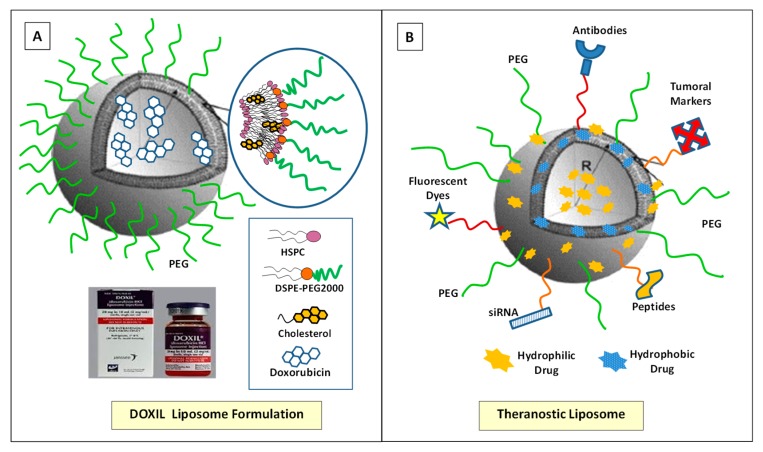
Main characteristics of the anticancer drug Doxil (**A**). Liposomes functionalization with various ligands, (peptides, fluorescent dyes, antibodies, aptamers, tumoral markers) and improvement of the specificity of the liposome interaction with target tissues (targeted drug delivery) (**B**).

Liposomes structural features may be strongly influenced by the presence of inclusion compounds (such as drugs or nanoparticles), and their presence may induce important structural perturbations against cohesive tendency of the liposomes bilayers structure [164,165,166,167,168]. The encapsulation of active agents within the bilayer structure, while favoring the drugs solubilization in aqueous media, also provides preservation and control against the degradation of the drug. Due to these properties, the employment of the liposome nanocarrier produces a sensitive improvement of the toxicity profiles and therapeutic efficacy. Owing to their easily controllable properties (such as the modulation of size, hydrophobic or hydrophilic character, biocompatibility and low toxicity), liposomes are considered the largest class of clinically-approved anticancer nano-formulations for therapeutic drugs [169,170]. Several liposome-based nano-formulations of anti-cancer agents (including Daunorubicin, Doxorubicin, Paclitaxel, Vincristine and Cisplatin) are currently in clinical use for cancer treatment [169,170]. In Figure 14A we report the main characteristics of the anticancer drugs Doxil, the first FDA-approved nanocarrier, consisting of a PEGylated liposomal doxorubicin nanocarrier for the clinical treatment of the epithelial ovarian Kaposi’s sarcoma [171].

The liposome PEGylation, i.e., their functionalization of with polyethylene glycol (PEG), favors an efficient drug delivery process, by improving the blood-circulation time and the colloidal stability of active therapeutics [172,173]. For this reason, in the last decades, the PEGylation process has become a standard in drug/nanocarrier functionalization for the development of advanced drug delivery systems [172,173]. However, a number of investigations has reported unexpected adverse immune responses such as the rapid clearance of PEGylated nanocarriers upon repeat administration, a phenomenon called accelerated blood clearance (ABC) [174]. In fact, the patients who undergo a treatment with PEGylated nanocarriers may stimulate the formation of antibodies that recognize (and bind) to PEG (i.e., anti-PEG antibodies) [174]. Therefore, patients that acquire anti-PEG antibodies exhibit an accelerated blood clearance and a lower drug efficacy [174,175].

Another adverse immune response consists in the adverse reactivity (hypersensitivity), which is correlated with a side effects of PEGylated nanocarriers, referred to as complement activation-related pseudoallergy (CARPA) [175]. These adverse immune responses require the development of alternative (synthetic, natural, or zwitterionic) polymers that are able to reproduce the desired functions and properties of PEG. In this respect, polyaminoacids, polyacrylamides, polivinilpirrolidone (PVP), zwitterionic polymers, polysaccharides, Poly(N-(2-hydroxypropyl)methacrylamide)s (PHPMA) and Poly(oligo(ethylene glycol) methyl ether methacrylate (POEGMA) are among the most promising candidates, which exhibit comparable (or superior) properties to PEG [175]. However, a deeper investigation is necessary to confirm their therapeutic benefits and their non-immunogenicity before their employment in clinical applications.

Liposomes can also be used for the controlled delivery of active drug molecules to specific sites (tumors or diseased tissues). The functionalization of the liposomes surface with different ligands, such as peptides, monoclonal antibodies, aptamers, growth factors, sensitively ameliorates the specificity of the liposome interaction with target sites (targeted drug delivery) [176,177]. Folate receptor (FR) is one of the most commonly employed tumor-associated antigens that can bind (with high affinity) with folic acid (FA) and its conjugates and is able to ingest the bound molecules inside the targeted cells via the endocytic pathway [178]. Folate ligands have been largely employed for the delivery, to FR-overexpressed cells, of different anticancer drugs (including Doxorubricin, Methotrexate, Vincristine, Paclitaxel), up to large macromolecules containing DNA, which proved to be highly effective as targeted drug delivery system [178]. For example, folate-conjugated liposomes evidenced an efficient internalization (though FR-α-mediated endocytosis) in H1299 cells. The induced apoptosis together with cell-cycle arrest favored a sensitive growth inhibition process [178].

Furthermore, liposome drug delivery processes can be triggered by *external stimuli*, (such as light, magnetic field, heat (hyperthermia) and ultrasound) or *internal stimuli* (such as pH, enzymes, and redox) [5]. Finally, liposomes can be designed with the ability to simultaneously conjugate cancer-targeting molecules (active targeting) for therapy treatment and diagnostic tasks (theranostics liposomes) (Figure 14B) [176,177]. The development of multi-component and multi-functional nanoplatforms allows to perform (within the same liposomal nanostructure) a variety of functions, such as the combined delivery of therapeutic agents, stimuli responsive assembly/disassembly processes and/or site-specific imaging and targeting capabilities (Figure 14B). However, additional investigations concerning the nanotoxicity issues and regulatory guidelines need to be resolved before the translation of liposome theranostics in the clinic.

### 5.3. Self-assembly of Oligonucleotides (DNA and RNA)

**Figure 15:**
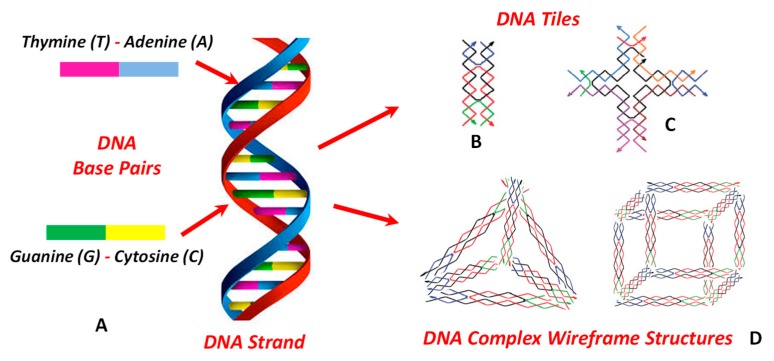
Schematic drawings of base pair composition and structure of the DNA strand (double helix) (**A**). Structure and morphology of the generated DNA complexes (DNA double crossover (DX) tile (**B**), 4 × 4 tile (**C**) and complex wireframe structure (**D**)). Colored lines represent different oligonucleotide strands with arrowheads.

In the last decades, DNA and RNA oligonucleotides have emerged as intriguing molecular building blocks, due to their ability to create highly functional biocompatible nanostructured architectures with accurate control of the structural properties [179,180]. The oligonucleotides self-assembly approach is based on the circumstance that two strands of DNA can bond together by tuning the non-covalent interactions at each complementary base pair of nucleotide. Each secondary DNA multi-tiles with specific properties can further self-assemble to form higher-order, complex nanostructures (Figure 15) [179,180]. Moreover, DNA tiles can be programmed according to specific instructions and algorithmic rules that allow a precise control of the successive layer of tiles. This approach allows a control of the evolution of the self-assembly process towards the formation of more complex DNA supramolecular nanostructures [181]. Moreover, complex DNA assemblies can be obtained by employing long strands that act as nucleation units for the ligation (at the corresponding strands) and the generation of high-order nanostructured functional systems (nucleated DNA self-assembly) [181,182]. The ability to program the nanostructures on the basis of initial sequences/conformations of strands allows the development of precisely designed nanomaterials. Using dedicated software, it is possible self-assemble highly functional nanostructures with controlled size, morphology, and desired functional moieties, which can be tuned for ligand attachments (at the desired sites). The precise engineering of the DNA building blocks represents a promising tool for the formation of various biotechnology applications such as intracellular delivery, molecular robotics, enzymatic nanoreactors, molecular lithography [180,181,182].

**Figure 16:**
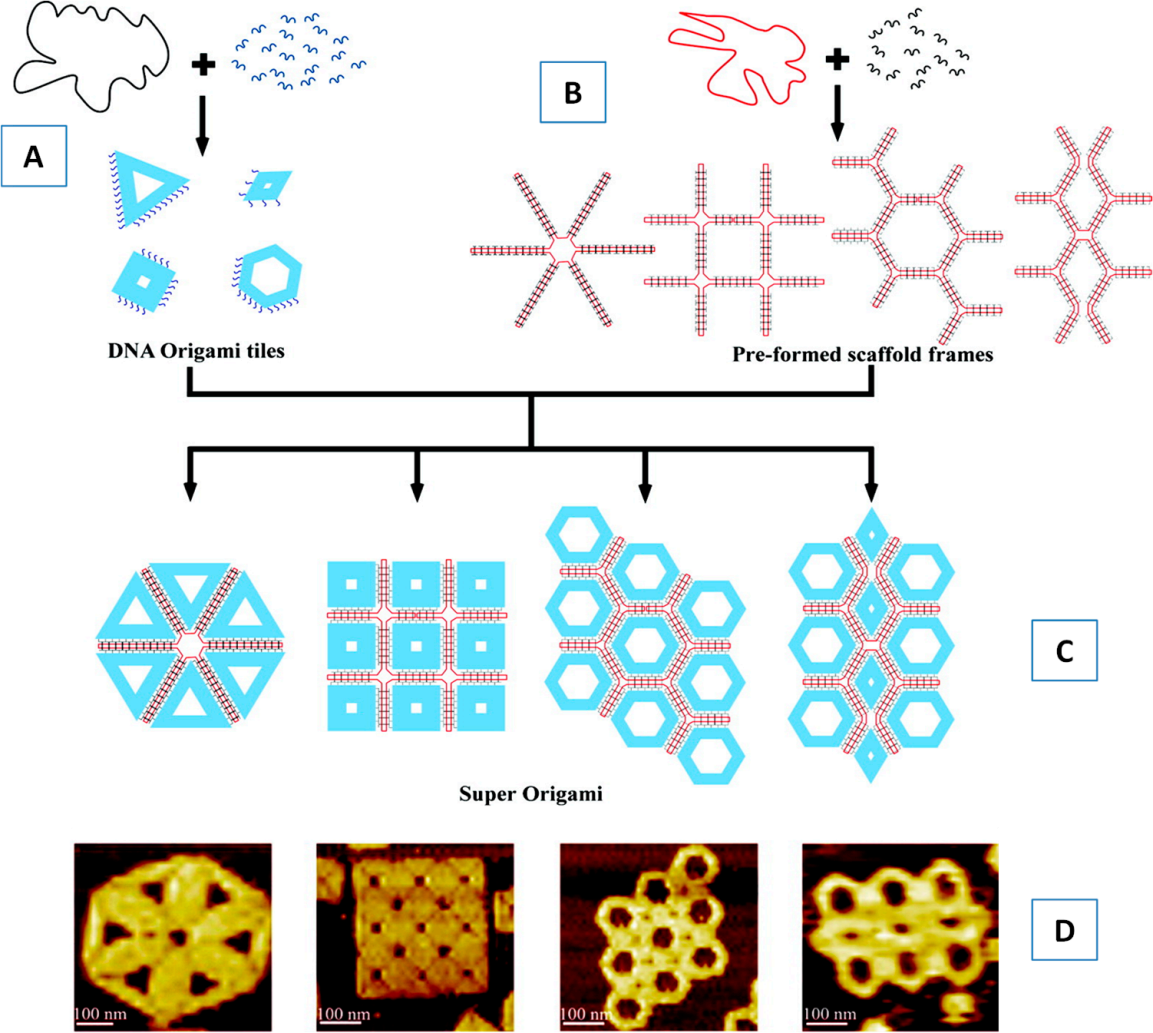
Mechanism of formation of superorigami. Initially, single stranded DNA scaffold (black circular strand) is pre-folded by a set of short DNA staples (blue bridge strands) to form various shapes of DNA origami tiles, (**A**). Moreover, some DNA scaffolds (red circular strand) are folded by a set of bridge strands (black strands) to form some tailored frameworks (preformed scaffold frames) (**B**). Finally, the preformed DNA origami tiles (that displays a group of single-stranded extensions, used as sticky points) are able to interact with the preformed scaffold frames to create the various super-origami structures (**C**,**D**). Adapted with permission from Reference [187]. Copyright (2011) American Chemical Society.

Furthermore, the self-assembly approach known as scaffolded DNA origami [182,183,184] represents a powerful fabrication strategy that allows the control over the system morphology. With the introduction of the DNA origami technique, it became possible to rapidly synthesize almost arbitrarily shaped molecular nanostructures at nearly stoichiometric yields. The technique furthermore provides absolute addressability in the sub-nm range, rendering DNA origami nanostructures highly attractive substrates for the controlled arrangement of functional species such as proteins, dyes and nanoparticles [185,186]. In fact, the size of the DNA origami tiles is limited by the scaffold strand length. A recent novel approach allows for the construction of “origami of origami”, or *“superorigami”*, starting from the formation of preformed DNA scaffold frameworks [187] (Figure 16).

Initially, a (single stranded) DNA scaffold is pre-folded by a set of short DNA staples (bridge strands) to form a DNA origami tiles. Some preformed scaffold frames, obtained by DNA scaffolds folded by a set of bridge strands, furnish the structural skeleton for the successive self-assembly. The preformed DNA origami tiles (that have a group of single-stranded extensions, used as sticky points) will serve as a templating staple during their interaction with the preformed scaffold frames. This strategy allows DNA origami nanostructures to grow into larger architectures.

DNA nanotechnology presents interesting applications also in the field of nano-mechanical measurement platforms [188] and molecular motors [189,190]. Controlled motion at the nanoscale can be achieved by using DNA base-pairing to direct the assembly and operation of a molecular transport system consisting of a DNA track (100 nm long) on a two-dimensional scaffold, a motor and fuel, all made from DNA [189]. The DNA motor loaded at one end of the track moves autonomously and at a constant (average) speed along the full length of the track. This long-range transport nanoplatform could lead to the development of precisely controlled systems (over both assembly and movement) that could be programmed by instructions encoded in the nucleotide sequences of the track and motor. The precise control allows the development of integrated systems (that combine active transport and information processing) for autonomous molecular manufacturing systems (assembly lines) or to fabricate molecular modelled (synthetic) ribosomes [189]. Moreover, a synthetic DNA-based system that integrates information processing and long-range transport (with a programmable path by using controlled instructions) has been recently developed [190]. When controlling by external instructions, about 87% of the motors follow the correct path, that decrease to about 71% with internal instruction control (carried by the motor itself) [190]. Programmable motion will allow the development of computing networks, molecular systems that can sort and process cargoes according to instructions that they carry, and assembly lines that can be reconfigured dynamically in response to changing demands [190].

The application of DNA nanotechnology is particularly interesting in the field of drug delivery and nanomedicine. Recent trends show, in fact, the possibility to develop molecular engineering strategies for the precise designer of devices (based on nucleic acid scaffolds) that are able to perform complex tasks such as the targeting cells (smart drug-delivery vehicles) and triggering the cellular actions/response in the biological environment (cell), or even whole living system [191,192,193,194].

Recently, a programming 20–30 nm (rectangular) DNA origami have been developed for loading and delivery of Doxorubicin drug by penetrating the ovarian cancer cells [194]. Cell penetrating investigations evidenced that the designed DNA origami are able to efficiently penetrate ovarian cancer cells and load the drug doxorubicin. Although further improvement of the nanocarrier colloidal stability is needed. The designed DNA origami shows promising applications in anti-tumor drug delivery in vivo [194].

However, the exploitation of the DNA origami technologies has been hindered by the difficulty of a large-scale fabrication process. This critical issue might be solved by utilizing bacteriophages (to generate single-stranded DNA precursor) thus allowing the cost-effective and scalable production of single DNA strands of virtually arbitrary length/sequences [195,196].

The integrity of DNA-based scaffolds can be compromised by physiological or cell-culture (salt concentrations) conditions that cause denaturation and additional degradation mediated by nucleases. For this reason, in order to enhance the structural and colloidal stability of (higher-order) DNA origami assemblies, several stabilization approaches have been recently developed, mainly based on the insertion of protecting (and coating) compounds [197,198,199,200] or by means of reversible covalent (stacking-bond contacts) stabilization [201,202].

Particularly interesting are the peptide–oligonucleotide conjugates (POCs), obtained by connecting a covalent bond with an oligonucleotide (such as a DNA molecule) to synthetic peptide sequences [203]. These building blocks combine the structural programmability of DNA/RNA oligonucleotides with the characteristic functionality of polypeptides. DNA scaffolds that are modified with (multiple) peptides might recapitulate the full-length (and functions) of proteins sequence, thus allowing the formation novel structures that are difficult to obtain with native proteins [203]. This allows the formation of new nanostructures that contain functional peptides sequences that are able to support some specific bioactivity or that have catalytic active sites [203,204]. Moreover, protein detection using DNA labelling can enhance the immunoassays sensitivity during the detection of biomarkers of specific proteins. With the development of immune-polymerase chain reaction (IPCR) (i.e., antibody-DNA conjugates employed for specific sensitive antigen detection), it is possible to analyze very small concentrations of specific markers levels with a sensitivity of some orders of magnitude below than conventional enzyme-linked immunosorbent assay (ELISA) protocol. This allows the amelioration of clinical processes and protein biomarkers for personalized medicine applications [205]. Moreover, a method for ultrasensitive detection of the biomarker’s microRNA (for the detection of disease such as the cellular differentiation, tumor development and immunological response) has been recently proposed. In this case the hybridizations between microRNA, DNA tetrahedron and primer probe initiate a rolling circle amplification on a DNA tetrahedron decorated electrode surface [206]. This approach has demonstrated an enhanced binding recognition efficiency at the electrode surface together with a sensitively improved electrochemical signals with a limit of detection of 50 aM (attomolar) of miRNA amounts, with a discrimination ability of a single base [206].

While DNA represents the pioneer building material in oligonucleotides nanotechnology, RNA emerges as an important alternative with its own advantages for nanomaterials self-assembly [207]. Despite the many common features between RNA and DNA, RNA presents generally a lower stability than DNA, and a higher probability of RNA strands to form undesired secondary structures [207,208]. On the other hand, RNA exhibits the ability to be encoded into genes and to be expressed in vivo. RNA nanostructures self-assembly relies mainly on single-stranded building blocks, and their ability to fold into a variety of predictable stable tertiary structures (such as the “kissing loops”) that can be used to produce, functional complex nanostructures [207,208]. Depending on their shape, size, and sequence, RNA nanoparticles can exhibit a strong immunogenicity that can be exploited in drug delivery applications or as adjuvants/reagents for immunotherapy treatments [207]. RNA can be used as a functional scaffold, as in the case of ribozymes, aptamers and siRNA [209]. The suitable spatial arrangement and control of the functional RNA domains allow the development of promising applications in RNA-based therapeutics and nanomedicine [209]. Moreover, self-assembly of RNA building blocks allows the construction of a variety of 2D/3D/4D nanostructures (such as polygons, filaments and arrays) for use in tissue engineering, biosensing [209]. Finally, the co-transcriptional RNA self-assembly, that exploits the coupling of RNA synthesis inside cells (by means of internal transcription machinery) with RNA self-assembly in vitro or in vivo allows the development of nanorings, nanocubes and organelle-like RNA scaffolds [209].

## 6. Emerging Technologies: Self-assembly of Carbon-based Nanostructured Materials

**Figure 17:**
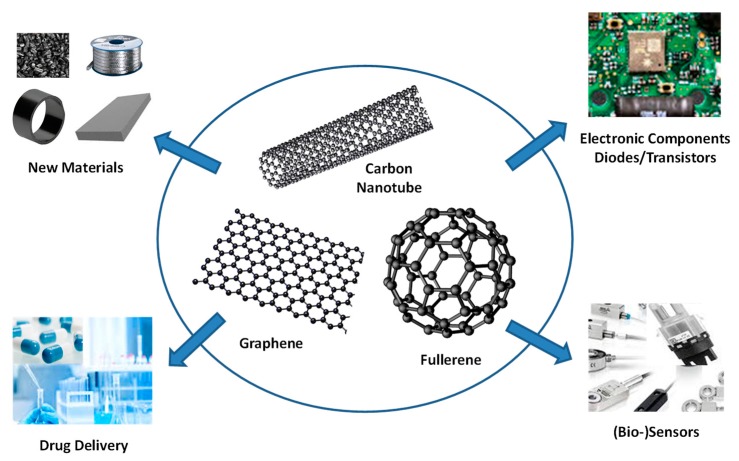
Morphology and main applications of some carbon based nanoplatforms (carbon nanotubes, graphene, fullerene) for emerging nanotechnologies.

Among the most important organic nanomaterials there are three major types of carbon-based nanostructured materials that play a significant role for the development new nanotechnologies, namely; carbon nanotubes (CNTs), graphene and fullerene (Figure 17). Owing to their high aspect ratio, good mechanical properties, excellent optical activity and multifunctional surface properties, these nanomaterials represent important nanoplatforms for the development of emerging nanotechnologies.

### 6.1. Carbon Nanotubes

Carbon nanotubes (CNTs) consist of one (or more) graphene sheets rolled up to form a tube-like single walled (SW-CNT) or multi-walled (MW-CNT) carbon nanotube structure [183]. The high aspect ratio, high conducting and mechanical properties and easy of functionalization of the surface endgroups represent only some of the important properties of carbon nanotubes that have been utilized for the design field-effect transistor and for the sensing of sugars, biomolecules and antibody–antigen complexes [210].

Depending on the specific nano-device application, it is desirable to drive the self-assembly process in order to control the place, orientation and morphology of the nanotube building blocks (on the nanometer length scale), in order to be able to pattern SW-CNTs as individual tubes, small bundles or thin films. Previous investigations evidenced that carbon nanotubes can be positioned, bent or welded (with nm accuracy) by using scanning probe instruments [211]. Other self-assembly methods, such as Langmuir–Blodgett methods, electrospinning, external field driven routes and DNA nano-templates have been also investigated for nanotube self-assembly [211].

**Figure 18:**
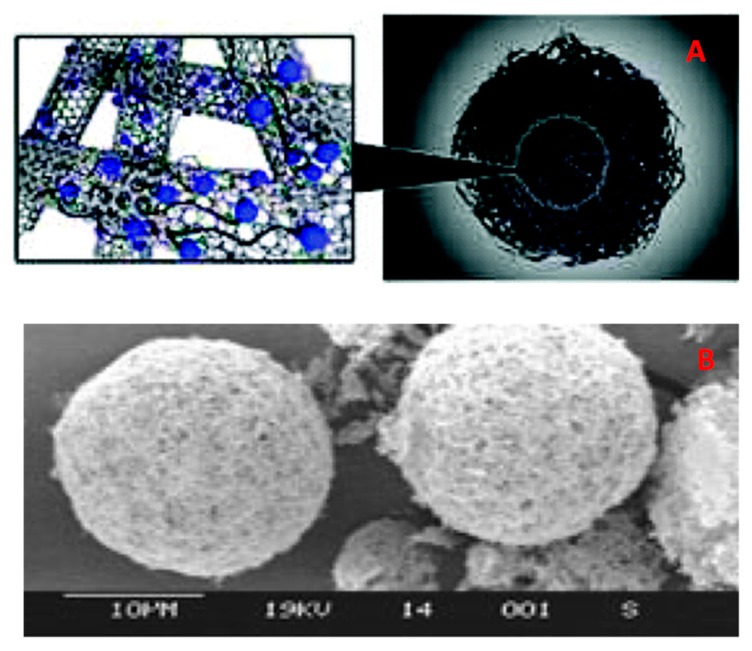
SEM image of (surfactant-mediated) self-assembled carbon nanotubes (CNTs) microspheres (**A**). Adapted with permission from ref. [212]. Copyright (2011) American Chemical Society. Self-assembly of CNTs nano-capsules from acid-modified multiwalled CNTs obtained by the water-in-oil (W/O) microemulsion approach (**B**). Adapted with permission from ref. [213]. Copyright (2007) American Chemical Society.

An interesting approach consists in the surfactant assisted self-assembly process of CNTs. The study of Zuber et al. [212] reports the formation surfactant-mediated CNTs microspheres through the entanglement of carboxylic acid-terminated multiwalled carbon nanotubes (CNTs) into spherical nano-assemblies. The SEM results (Figure 18A) revealed that the surfactant-wrapped CNTs microspheres were densely packed with relatively smooth exterior surfaces. The self-assembly of CNTs microspheres, with a high range of diameters and porosities and large reactive areas, can offer a new versatile nanoplatform to large variety of advanced applications [212]. Moreover, Song et al. [213] proposed a self-assembly approach of CNTs nano-capsules (diameter of 5−10 μm) from acid-modified multiwalled carbon nanotubes (CNTs) using a water-in-oil (W/O) microemulsion method (Figure 18B). In that case, it was found that the dimension of the CNTs capsules was strongly dependent on the amount of CNTs in water and the length of CNTs [213].

Of special interest is the use of CNTs self-assembly in bio-medical applications. Single-walled carbon nanotubes (SW-CNTs) are able to deliver imaging agents, (fluorophores or radioisotopes) or drugs to target tumors and offer sensitive advantages over the approaches based on use of the antibodies or other nanoplatform [214,215]. In particular, the CNTs can incorporate a high quantity of active agents (100 times higher than with a monoclonal antibody), and due to their high aspect ratio, can be eliminated rapidly from the circulation by the renal filtration, thus mitigating the radioisotope toxicity [213]. Moreover, carbon nanotubes have been utilized as efficient nanoformulation for drug delivery and gene therapy applications [210,211]. More specifically, the aromatic surface of SW-CNTs favors the (supramolecular) π–π stacking interactions with the aromatic drugs, such as the doxorubicin (DOX). Higher loading capacities of CNTs were obtained compared to liposomes or micelles, while the DOX-loaded SW-CNTs exhibit an enhanced therapeutic anti-tumoral effect and less toxicity than the free DOX. Recently, a novel pH-responsive and targeting folic acid (FA)-modified MW-CNTs has been proposed for the delivery of the drug doxorubicin (DOX) to tumor sites [216]. The nanocarrier exhibited exceptional colloidal stability and high drug encapsulation efficiency (70.4%), while anticancer (in vivo) experiments evidenced that the MW-CNTs nanocarriers enhance the suppression of tumor growth and decrease the side effects connected with free DOX [216].

Finally, the CNTs applications in the field of microelectronics are particularly interesting. Engel et al. [217] investigated the formation of thin film transistors (TFT) based on self-assembled, aligned, semiconducting carbon nanotube arrays (CNT-TFT) [217]. In that case, the films were formed from a solution of pre-separated (99% purely) semiconducting CNTs that self-assemble into micron-wide strips of arrays of highly aligned CNT films that cover the whole nano-device (chip). The assembled CNT-TFTs were both photo- and electroluminescent and delivered strong photocurrents (exhibiting both high drive currents and large on/off current ratios) [217].

### 6.2. Graphene

Graphene is composed by a single layer of carbon atoms arranged in a honeycomb-like lattice [218]. Due to its two-dimensional sp^2^-bonded structure, it possesses exceptional mechanical, thermal, optical and electronic properties. One of the most promising utilizations of graphene nanostructures is its applications as an ultrasensitive chemical or gas sensor [219]. Due to their special physico-chemical characteristics and electronic properties, the graphene can yield various applications, including batteries, light emitting diodes and supercapacitors [220,221]. Moreover, biomedical applications of graphene include bio-medical imaging and anticancer drug delivery [222,223]. Recently, graphene oxide (GO) nanocarriers functionalized with lactosylated chitosan oligosaccharide was constructed for the targeted drug delivery of doxorubicin drug and DNA sequences into the (human) hepatic carcinoma cells (QGY-7703) [223]. The maximal loading capacity of the drug and fluorescein amidites labeled DNA (FAM-DNA) reached 477 µg/mg and 4 mmol/g, respectively. The nanocarrier was able to deliver the complex to the specific site (i.e., QGY-7703) for over 30 min, while no significant toxicity has been detected, even at the high concentration of 100 μg/mL [223].

Graphene self-assembly represents a promising method also for microelectronic applications. Recently, graphene micro-patterns (consisting of crossed stripe of single- and two-layer graphene) have been fabricated by means of the (evaporation-induced) self-assembly technique [224]. Based on these graphene micro-patterns, graphene-based field effect transistors (G-FET) have been engineered, which operates at low voltages (<2 V) and with a hole and electron mobility of 214 and 106 cm^2^/V·s, respectively. The proposed self-assembly method paves the way for the (non-lithographic) engineering of versatile graphene nanoplatforms on flexible substrates with a simple, cost effective and scalable approach [224].

### 6.3. Fullerene

**Figure 19:**
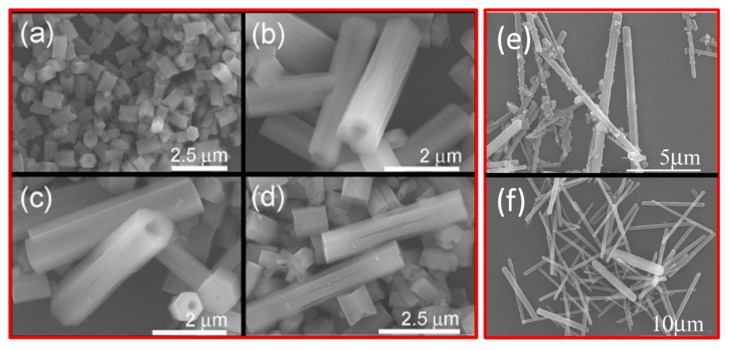
SEM images of fullerene C_60_ assemblies (tubes and rods) growing at very early stages. Initially, the C_60_ self-organize into nanoparticles, when the formation process of C_60_ 1D structure is stopped by fast diluting the system into abundant ethanol (**a**). C_60_ tubes growing at two seconds after the self-assembly initiation (**b**–**d**). Sample of the C_60_ (concentration of 1.0 mg/mL and isopropanol/m-xylene volume ratio of 1:1) obtained in the absence of surfactant (showing irregular rods and small seeds) (**e**), and with the surfactant cetyltrimethylammonium bromide (CTAB) at concentration of 0.5 mM (showing rods with poor uniformity) (**f**). Adapted with permission from Reference [229]. Copyright (2007) American Chemical Society.

Fullerenes consist in closed hollow cages of carbon atoms interconnected in pentagonal and hexagonal rings [225,226]. Due to their hydrophobic nature fullerenes is insoluble in water, while its functionalization using carboxyl, amino, or hydroxyl groups allows the enhancement the water solubility and result in higher biocompatibility. Fullerenes find application in the field of organic photovoltaics (OPVs), especially in the power conversion efficiency of the devices, in the development of more efficient plastic solar cell structure, and in fuel cells nanotechnology [227,228]. Fullerene-based nanomaterials can be prepared, in a controllable manner, by means of the self-assembly process in presence of surfactants. Figure 19 reports the sub-micrometer single-crystal nanoaggregates (rods and tubes), obtained by Ji at al. [229], during the (solvent-induced) self-assembly of C_60_ fullerenes (assisted by the surfactant CTAB in an isopropanol/m-xylene solution). The investigation evidenced that the length (and length-to-width ratio) of the single-crystal rods and tubes were found to be dependent on the concentration of C_60_ in the stock solution. At the fixed concentration of 1.2 mg·mL^−1^, increasing the volume ratio of isopropyl alcohol (IPA) and C_60_ solution in m-xylene (from 1:1 to 1:3) resulted in a shape transition from rod to tube [229]. This proposed fullerenes self-assembly method can have potential applications in the development of miniature devices.

Due to their hydrophobic surface and their optimal sizes that facilitate an easy crossing of cell bio-membranes, functionalized fullerenes can be employed as efficient nanoplatforms for drug and gene delivery [230].

Recently, a fullerene-based drug nanocarrier systems for doxorubicin drug was developed for potential lung cancer therapy [231]. Moreover, fullerene derivatives with well-defined functional characteristics are promising nanostructures for the preparation of bio-active macromolecules with optimal bio-distributions. Some studies have shown that stable DNA-C_60_ hybrids are capable of effective cell transfection, with distinct organ selectivity [232]. These investigations evidenced that appropriate hydrophilic–lipophilic balance is crucial for the formation of DNA–fullerene complexes for effective gene transfection [232].

Finally, the fullerene self-assembly can be employed for the development of novel detectors. In a recent investigation, Wei et al. [233] performed a controlled self-assembly of C_60_ (assisted by the solvent carbon bisulfide CS_2_) into single-crystal ultrathin (_2_C_60_·_3_CS_2_) micro-ribbons. The formation of π–π stacking 1D chains of C_60_ (during the nucleation period) was favored by the formation of a surrounding CS_2_ cage-like structures, which acted as glue and favored the lateral assembly of the 1D C_60_ chains into ultrathin 2D micro-ribbon single crystals. These self-assembled fullerene microribbons, that exhibit highly sensitive photoconductivity properties in a spectral range covering ultraviolet (UV) to visible, can be employed for the development of advanced photodetection systems [233].

## 7. Nature-Inspired Nanomaterials: Self-Assembly of Nanostructured Dyes for Solar Cells Applications

Self-assembly processes have been recently employed for the construction of dye-sensitized solar cells (DSSCs). DSSCs represent a new class of solar cells, which are developed by the imitation of the photosynthesis process, by using low-price abundant materials, such as TiO_2_, sensitizers and simple manufacturing technology [234,235]. Since Gratzel and co-workers [234], many researchers have used the self-assembly approach for the design of many new nanostructured dyes and new sensitization methods for the development of DSSCs [236,237,238,239,240]. These approaches employ a series of new self-assembly strategies that eliminate expensive synthesis steps and realize a high yield [241,242,243,244,245]. Environmentally friendly and non-toxic vegetable dyes such as carotenoids, betalains, anthocyanins and chlorophylls (extracted from fruits, leaves and flowers) have been recently proposed as eco-compatible sensitizers in DSSCs [246].

Recently, two different organic antenna chromophores (S1, S2) coordinated via zinc with two acceptors (A1, A2) were prepared to construct an A−Zn−S series of self-assembled dyes for solar cells applications [247]. The self-assembly approach, obtained by coordination with some organic antenna chromophores, allows for avoiding the complex synthesis of traditional dyes. The approach allows the construction of nanostructured materials with a light harvesting improved ability with a charge recombination process that can be simply reduced by modifying the structures of the antenna chromophores and acceptors [247]. Finally, self-assembled ruthenium (II), phthalocyanine (RuPc) and N-pyridyl-peryleneimide (PyPMI) dyad based on a noncovalent interaction has been recently used for the construction of n-type DSSCs [248].

These studies demonstrate, once again, that the supramolecular design based on non-covalent interaction between self-assembled building blocks represent an alternative approach to replace the most expensive synthesis of covalent-linked macromolecular systems.

## 8. Conclusions and Future Perspectives

We highlighted recent advances of the self-assembly processes employed for the development of new functional platforms for nanomaterials and biomaterials applications. The building block properties (size, morphology, surface functionality), the mutual and cooperative interactions, as well as the initial spatial configuration (and environment conditions) play a crucial role in the design of efficient nanostructured materials and have a great impact on the evolution towards the desired functional products. The organic materials (building blocks) present better characteristics and properties to match the physico-chemical condition encountered in biological tissues and represent the best examples of biocompatible nanostructures. However, despite the variety of smart biomaterials developed in recent years, their effective therapeutic use is often hindered by the complexity of the encountered biological environments that strongly influence the effective functionality of the nanomaterial. Therefore, the identification of the fundamental factors involved in the hierarchical self-assembly of nanostructures represents the initial step that drives towards the development of novel protocols for the construction of novel advanced functional materials.

## Figures and Tables

**Table 1:** Strength of the main interactions involved in nanomaterials self-assembly.

Bonding and Interaction Type	kJ/mol
Covalent bond	100–400
Van-der-Waals interaction	<5
Hydrogen bond	4–120
Hydrophobic effects	Entropy
π–π interaction	0–50
Metal–Ligand	0–400
